# Adhesive Fiber Stratification in Uropathogenic *Escherichia coli* Biofilms Unveils Oxygen-Mediated Control of Type 1 Pili

**DOI:** 10.1371/journal.ppat.1004697

**Published:** 2015-03-04

**Authors:** Kyle A. Floyd, Jessica L. Moore, Allison R. Eberly, James A. D. Good, Carrie L. Shaffer, Himesh Zaver, Fredrik Almqvist, Eric P. Skaar, Richard M. Caprioli, Maria Hadjifrangiskou

**Affiliations:** 1 Department of Pathology, Microbiology & Immunology, Vanderbilt University School of Medicine, Nashville, Tennessee, United States of America; 2 Department of Chemistry, Vanderbilt University School of Medicine, Nashville, Tennessee, United States of America; 3 Department of Chemistry, Umeå University, Umeå, Sweden; 4 Umeå Center for Microbial Research, Umeå University, Umeå, Sweden; 5 Department of Biology and Department of Chemistry, Belmont University, Nashville, Tennessee, United States of America; 6 Department of Medicine, Vanderbilt University School of Medicine, Nashville, Tennessee, United States of America; 7 Departments of Biochemistry and Pharmacology, Vanderbilt University School of Medicine, Nashville, Tennessee, United States of America; University of Washington, UNITED STATES

## Abstract

Bacterial biofilms account for a significant number of hospital-acquired infections and complicate treatment options, because bacteria within biofilms are generally more tolerant to antibiotic treatment. This resilience is attributed to transient bacterial subpopulations that arise in response to variations in the microenvironment surrounding the biofilm. Here, we probed the spatial proteome of surface-associated single-species biofilms formed by uropathogenic *Escherichia coli* (UPEC), the major causative agent of community-acquired and catheter-associated urinary tract infections. We used matrix-assisted laser desorption/ionization (MALDI) time-of-flight (TOF) imaging mass spectrometry (IMS) to analyze the spatial proteome of intact biofilms *in situ*. MALDI-TOF IMS revealed protein species exhibiting distinct localizations within surface-associated UPEC biofilms, including two adhesive fibers critical for UPEC biofilm formation and virulence: type 1 pili (Fim) localized exclusively to the air-exposed region, while curli amyloid fibers localized to the air-liquid interface. Comparison of cells grown aerobically, fermentatively, or utilizing an alternative terminal electron acceptor showed that the phase-variable *fim* promoter switched to the “OFF” orientation under oxygen-deplete conditions, leading to marked reduction of type 1 pili on the bacterial cell surface. Conversely, S pili whose expression is inversely related to *fim* expression were up-regulated under anoxic conditions. Tethering the *fim* promoter in the “ON” orientation in anaerobically grown cells only restored type 1 pili production in the presence of an alternative terminal electron acceptor beyond oxygen. Together these data support the presence of at least two regulatory mechanisms controlling *fim* expression in response to oxygen availability and may contribute to the stratification of extracellular matrix components within the biofilm. MALDI IMS facilitated the discovery of these mechanisms, and we have demonstrated that this technology can be used to interrogate subpopulations within bacterial biofilms.

## Author Summary

Bacteria are commonly found in multicellular communities known as biofilms. Biofilms can form on a variety of surfaces, both outside and within living things, and can have detrimental effects on human health. The characteristics of bacteria occupying different areas within biofilms are not well understood, and such knowledge is critical for understanding how biofilms form and for developing strategies to treat biofilm-related infections. Here, we adapted a technique to sample how proteins cluster within bacterial biofilms as a means to identify the location of bacteria with differential protein expression within the community. We observed that with uropathogenic *E. coli*, which is the major cause of urinary tract and catheter-associated urinary tract infections, bacteria close to the air-exposed region of the biofilm expressed different adhesive fibers compared to those at the liquid interface. We went on to show that lack of oxygen shuts down the production of fibers known to be critical for adherence to host bladder cells and to catheter material. This discovery was enabled by a new application of an existing technology that allowed us to gain insights into the spatial regulation of proteins within bacterial biofilms and to elucidate pathways that could be targeted to inhibit bacterial adherence.

## Introduction

In nature, bacteria predominantly exist in a biofilm state [1] forming mutualistic or parasitic associations with other living organisms [2,3]. Within vertebrate hosts, the resident microbiota are essentially multi-species biofilms that play a key role in preventing colonization by pathogens [4]. Conversely, pathogenic bacteria exploit biofilm formation to colonize prostheses, catheters, as well as extracellular and intracellular host niches resulting in potentially life-threatening infections that are often difficult to treat [5]. Both single and multi-species biofilms are heterogeneous in nature, comprised of bacterial subpopulations with distinct tasks, such as expression of matrix components or a specific metabolic activity [6–9]. This “division of labor” within the community contributes to recalcitrance of the biofilm to antibiotic treatment. Biofilm subpopulations can be transient in nature, and arise in response to alterations in nutrient and oxygen availability of the surrounding microenvironment that in turn leads to local changes in bacterial gene expression [6–9]. However, little is known about the expression and distribution of individual protein species within a single multicellular community that results from this differential gene expression and how such differences may shape the characteristics and the fate of the biofilm.

Traditional techniques used to visualize protein distribution within intact biofilms rely on microscopy-based methods that require the use of either fluorescently labeled proteins or the application of antibodies specific to a protein of interest [10,11]. These techniques are limited to previously identified protein targets and can typically only accommodate one or two species in a single analysis. Conversely, more global genomic and proteomic-based analyses necessitate the destruction of biofilm architecture, leading to complete loss of spatial information.

**Fig 1:**
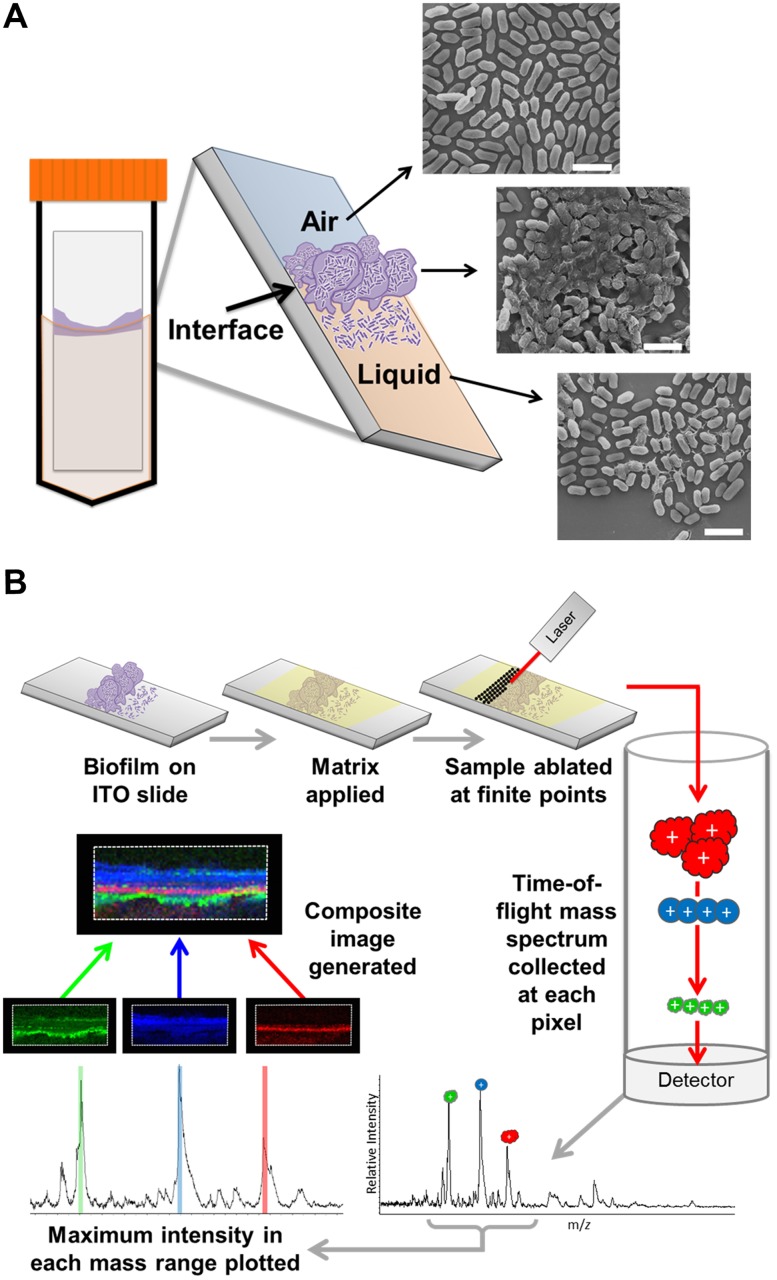
MALDI IMS as a tool to dissect the spatial proteome of bacterial biofilms. (A) Schematic depicting the culture method for single species surface-associated biofilms. Sterile ITO-coated borosilicate glass slides were placed into a 50 mL conical containing media seeded with bacteria, such that the air-liquid interface would constitute the center of the slide, and biofilms were cultured for 48 hours. The biofilms form at the air-liquid interface in response to the induced environmental and nutrient gradients created by the culture conditions, as indicated in the schematic of the resulting surface-associated biofilm and depicted by representative SEM micrographs of each region. Micrographs were obtained from a 48-hour ethanol-washed UPEC surface associated biofilm. Scale bar = 2.5 μm. (B) Description of the MALDI-TOF IMS pipeline as applied to the analysis of bacterial biofilms. Surface-associated biofilms were given an organic solvent wash to decrease lipids and salts from within the sample that interfere with protein ionization. The biofilm was then overlaid with an UV-absorbing matrix, and analyzed by MALDI IMS.

Matrix-assisted laser desorption/ionization time-of-flight imaging mass spectrometry (MALDI-TOF IMS) is a surface-sampling technology that can determine spatial information and relative abundance of analytes directly from biological samples [12]. Samples are treated with a matrix that absorbs ultraviolet light from a laser source to ionize analytes of interest. The generated ions are accelerated along a time-of-flight (TOF) mass analyzer for separation and detection [13]. Using this technique, spectra are collected in a defined array across the sample, and each peak intensity in the spectra is then extrapolated to generate an ion intensity map, allowing for a two-dimensional representation of analyte distribution within the imaged array (Fig. 1 and [14]). This label-free technology does not require prior knowledge of sample composition or analyte distribution and provides an unbiased approach for the simultaneous localization analysis for multiple analytes within a single biological sample.

Here, we used MALDI-TOF IMS to examine the *in situ* distribution and localization of low molecular weight proteins within biofilms formed by uropathogenic *Escherichia coli* (UPEC). UPEC, one of the extra-intestinal *E. coli* pathotypes and the primary cause of urinary tract infections, can form extracellular biofilms on host cells and urinary catheters, as well as intracellular biofilm-like communities within host bladder epithelial cells [15–19]. These UPEC virulence mechanisms dictate multiple disease outcomes [20], including urosepsis that can have life-threatening complications [21]. MALDI IMS detected distinct protein localization patterns within the surface-associated UPEC biofilms imaged in these studies. Subsequent, conventional proteomic approaches led to the identification of several of the distinctly localized ion species. Among the proteins identified were CsgA and FimA, which comprise the primary structural subunits of curli and type 1 pili fibers respectively. Type 1 pili, encoded by the *fim* gene cluster, are chaperone-usher pathway (CUP) pili [22] that facilitate adherence to mannosylated moieties and are the primary determinant that enables a) UPEC attachment to the bladder urothelium, and b) inter-bacterial interactions in both extracellular and intracellular biofilms [15,23].

MALDI IMS revealed that, while curli subunit signatures are found at the air-liquid interface of the biofilm, which is consistent with their primary role in extracellular matrix infrastructure, type 1 pili subunit signatures predominantly localize to the air-exposed regions of the biofilm. Subsequent studies investigating the effects of anaerobiosis on expression of type 1 pili in UPEC led to the discovery of two regulatory mechanisms controlling expression of type 1 pili in response to the presence of oxygen. Together, these data demonstrate how MALDI IMS can be used to dissect the spatial proteome of an intact bacterial biofilm, and highlight how the information obtained can provide new insight into protein regulation relating to biofilm infrastructure.

## Results

### Development of biofilm culture methods for MALDI-TOF IMS

In order to assess the utility of MALDI-TOF IMS for evaluating protein localization within bacterial biofilms, we adapted a simple surface-associated biofilm setup that enabled the sampling of single-species biofilms formed by uropathogenic *Escherichia coli* (UPEC) [24]. We optimized growth conditions to promote biofilm formation onto indium tin oxide (ITO) coated glass slides, given that our MALDI IMS must be performed directly from an electrically conductive surface for high voltage analyses [25]. Slides were placed vertically into culture media seeded with bacteria, such that only half of the slide was submerged within the media. This setup created an environmental gradient of oxygen and nutrients that induced biofilm formation at the air-liquid interface (Fig. 1A). We hypothesized that MALDI IMS would enable detection of distinct bacterial subpopulations resulting from the induced gradient (Fig. 1A).

MALDI-TOF IMS requires the application of a UV-absorbing matrix for analyte ionization [25] (Fig. 1B). Typical sample preparation methods begin with solvent washes to decrease ion suppression from lipids and salts within the sample in order to enhance protein ionization [25]. Here, we selected a sequential washing procedure of 70%, 90%, and 95% ethanol for 30 seconds each. Following washes, we evaluated biofilm integrity using three different techniques: crystal violet staining, scanning electron microscopy (SEM), and optical profilometry (S1 Fig). SEM analysis of the air-exposed, the air-liquid interface, and liquid-exposed regions of the biofilm indicated that the tertiary structure, along with cell shape and surface features, were preserved post-washing (S1 Fig). Crystal violet staining [26] and subsequent quantitation showed that the preparative ethanol washes did not significantly reduce biofilm levels (S1 Fig). Finally, optical profilometry [27] was used to assess the biofilm depth on the surfaces analyzed by MALDI IMS (S1 Fig). Combined, these approaches indicated that the sample preparation methods for MALDI IMS did not significantly perturb biofilm integrity.

### MALDI IMS reveals distinct protein localizations within surface-associated UPEC biofilms

**Fig 2:**
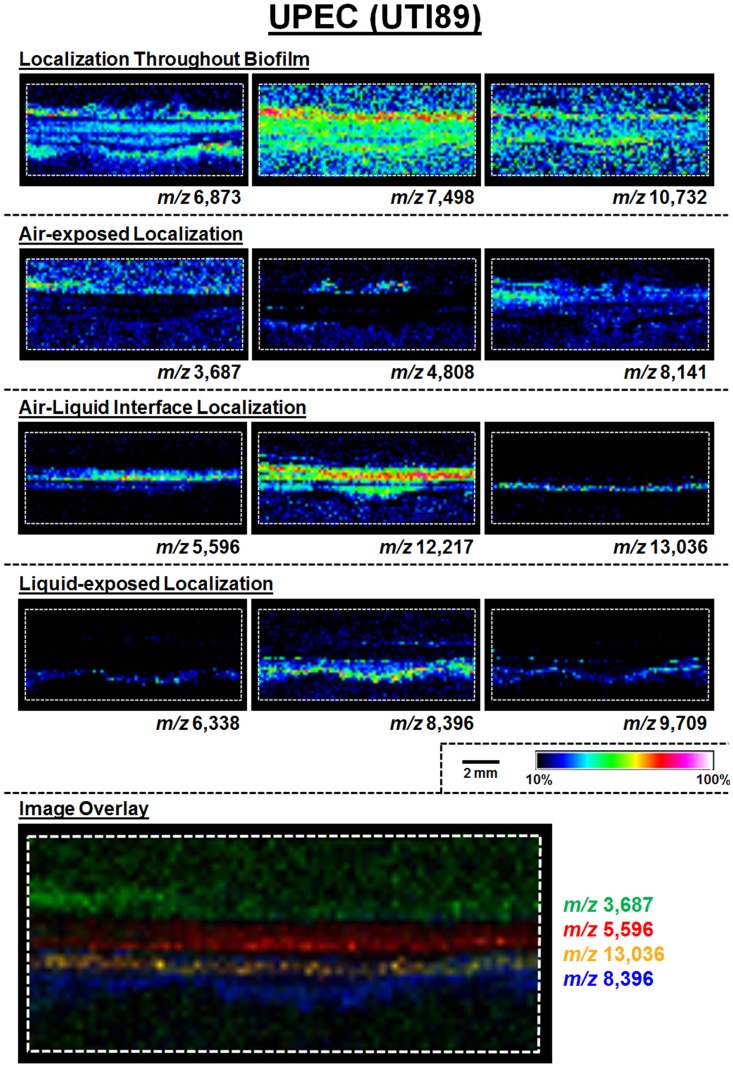
MALDI IMS reveals distinct protein localizations within UPEC biofilms. Representative ion images depicting distinct protein localization patterns observed in UPEC biofilms after 48 hours of growth. Images shown are from the same IMS analysis. Protein localizations for UPEC were validated in 16 biological replicates. The depicted mass-to-charge (*m/z*) ratio of each selected ion was determined after internal calibration of the total ion current-normalized average spectrum using mMass software [32]. Internal calibration used the theoretical mass, minus the signal peptide for proteins identified in the LC-MS/MS analyses to obtain the best mass accuracy for the data (as previously described [72]). Images are depicted ± 5 Da for each *m/z* species and data are presented as a heat map intensity of relative abundance from 10 (blue)– 100% (Red/White). Overlay images are presented using the same criteria, with single color distribution instead of a heat map from 10–100% intensity. Scale bar = 2 mm.

A schematic for the MALDI-TOF IMS analysis of UPEC biofilms is shown in Fig. 1B. The MALDI methods and matrix selected for these studies were optimized for lower molecular weight protein species; therefore, all analyses were carried out over an ion range of mass-to-charge ratio (*m/z*) 2,000–25,000. Within this range, we observed 60 UPEC protein ion species that were detected reproducibly in at least 5 biological replicates (S1 Dataset). The relative abundance and localization patterns for representative ion species are shown in Fig. 2. Each panel depicts a heat-map intensity plot for a unique ion species within the biofilm, where red/white indicates the highest levels of relative abundance, and black/blue the lowest levels (Fig. 2). All observed ion species displayed one of the following localization/distribution patterns: diffuse distribution throughout the biofilm, localization specific to the air-exposed or liquid-exposed region, or localization to the air-liquid interface (Fig. 2). Overlay analysis of ion images demonstrated that we could differentiate localization patterns for different protein species within the same region of the biofilm (Fig. 2, ion overlay of *m/z*’s 5,596-red and 13,036-yellow).

### Type 1 pili-producing and curli-producing bacteria occupy distinct regions of the UPEC biofilm

**Fig 3:**
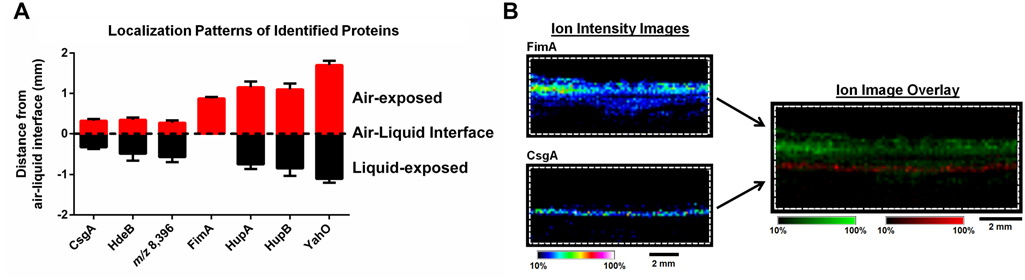
IMS analysis reveals stratification of identified UPEC proteins and distinct localization of FimA and CsgA within the biofilm. (A) Graphical representation of the localization of each protein identified in Table 1, along with the unidentified ion *m/z* 8,396. The localization of the major curli subunit, CsgA, was used to demarcate the air-liquid interface. Localization of ions were measured as the distance (mm) from the middle of CsgA localization to the middle of the localization of each individual ion using Fiji Image J software [73]. Localizations were plotted using GraphPad Prism version 6. (B) IMS ion images of FimA and CsgA localization. Images are depicted ± 5 Da for each ion, and data are presented as a heat map intensity of relative abundance from 10 (blue)– 100% (Red/White). FimA (green) and CsgA (red) ion overlay image presented using the same criteria, with single color distribution instead of a heat map from 10–100% intensity. Scale bar = 2 mm.

Following MALDI IMS spatial analysis, enzymatic digestion of biofilm lysates and tandem mass spectrometry were used to identify select ion species observed (Table 1). These analyses identified the histone-like global transcriptional regulators HU-α (UniProt KB Q1R5W6, *m/z* 9,535) and HU-β (UniProt KB Q1RF95, *m/z* 9,226), which co-localized throughout the biofilm and were most abundant in the air-exposed region (Fig. 3A and S2 Fig). The acid stress-response chaperone protein, HdeB (UniProt KB Q1R595, *m/z* 9,064), and the uncharacterized protein YahO (UniProt KB Q1RFK1, *m/z* 7,718) were also identified (Table 1). HdeB localized to the air-liquid interface and was most abundant towards the liquid-exposed surface, while YahO localized throughout the biofilm (Fig. 3A and S2 Fig). Finally, two of the IMS signals identified by proteomics corresponded to major subunits of two UPEC adhesive organelles: The major curli subunit CsgA (UniProt KB Q1RDB7, *m/z* 13,036), an essential determinant for UPEC biofilm formation under the culture conditions used for these studies [7,28], and; the major subunit of type 1 pili, FimA (UniProt KB Q1R2K0, *m/z* 16,269).

**Table 1:** Identified proteins observed by IMS within 48-hour UPEC biofilms.

Protein Name	UniProtKB Accession Number	Theoretical Average Mass (Da)	Predicted Signal Peptide (Da)*	Theoretical Mass Minus Signal Peptide (Da)	Observed Average Mass (Da)^#^
Adhesion					
Type 1 pili major subunit, FimA	Q1R2K0	18,553	aa1–23 / 2,302 Da	16,269	16,269
Major curli subunit, CsgA	Q1RDB7	14,992	aa1–20 / 1,974 Da	13,036	13,036
DNA-binding / Transcriptional Regulation					
DNA-binding protein Hu-α	Q1R5W6	9,534	n/a	9,534	9,535
DNA-binding protein Hu-β	Q1RF95	9,226	n/a	9,226	9,226
General Stress Response					
Acid stress-response protein, HdeB	Q1R595	12,522	aa1–33 / 3,475 Da	9,065	9,064
Uncharacterized					
YahO	Q1RFK1	9,929	aa1–21 / 2,240 Da	7,707	7,718

*Predicted signal peptides obtained using the SignalP Server.

^#^Observed average mass obtained from IMS analysis of one representative 48 hour UPEC biofilm. Internal calibration was performed with mMass Software using the theoretical mass minus the signal peptide for proteins identified by tandem mass spectrometry analyses to obtain the best mass accuracy from the data.

Based on the MALDI IMS results, CsgA signatures were predominantly found at the air-liquid interface of the biofilm (Fig. 3A-B and S2 Fig), consistent with the role of curli as the primary extracellular matrix (ECM) component under the biofilm conditions tested. Conversely, FimA localized uniquely to the air-exposed region of the biofilm (Fig. 3A-B, and S2 Fig). Under the biofilm growth conditions used for these studies, type 1 pili have been shown to play an accessory role to biofilm infrastructure, and loss of type 1 pili impairs integrity but does not abolish biofilm formation [7]. Thus, we took advantage of a *fim* deletion mutant (UTI89Δ*fimA-H*) to validate the identification of the *m/z* 16,269 ion as FimA. MALDI IMS analysis of UTI89Δ*fimA-H* biofilms showed a loss of the ion at *m/z* 16,269 (S3 Fig), confirming the ion *m/z* 16,269 as FimA. Similarly, the ions *m/z* 9,535 and *m/z* 7,718 were validated as HupA and YahO respectively, through MALDI analysis of UTI89 mutants lacking the respective gene (UTI89Δ*hupA* and UTI89Δ*yahO*) (S3 Fig).

**Fig 4:**
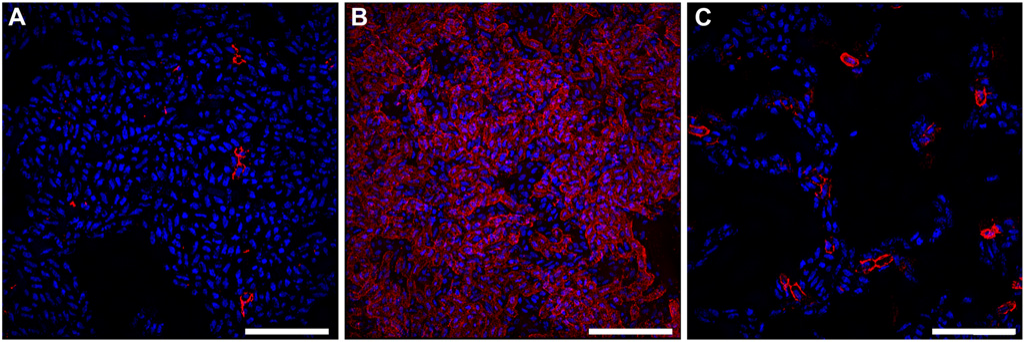
Bacteria expressing curli amyloid fibers localize primarily to the air-liquid interface of a 48-hour surface-associated biofilm. Representative immuno-fluorescence images obtained with super-resolution SIM microscopy from the (A) air-exposed region, (B) air-liquid interface, and (C) liquid-exposed region of the biofilm. Immuno-fluorescence was performed with DAPI staining for nucleic acid (Blue) and Alexa Fluor-555 conjugated secondary antibody detection of α-CsgA antibody (Red). Images shown are from a single biological replicate and are representative of two biological replicates total. A video depicting three-dimensional reconstruction of the biofilm at the air-liquid interface is provided in the supplemental material (S1 Video). Scale bar, 10 μm.

Given that curli are essential for UPEC biofilm formation under the conditions tested, we utilized a more traditional immuno-fluorescence approach with an antibody against CsgA to visualize curli-expressing bacteria within the biofilm and validate CsgA localization to the air-liquid interface. Combining immunohistochemistry with super-resolution structured illumination microscopy (SIM), we observed that the majority of curli-producing bacteria localized to the air-liquid interface of the biofilm, with only sparse populations found at the air- and liquid-exposed regions (Fig. 4, S1 Video). These data confirmed the IMS observations of CsgA localization to the air-liquid interface of the biofilm. As an orthologous approach, we took advantage of small peptidomimetic molecules that interfere with curli biogenesis in UPEC [29]. We hypothesized that treatment of pre-formed biofilm with one such compound, FN075 [29], should block curli fiber subunit incorporation leading to an abundance of CsgA monomers within the biofilm that could be detected by IMS. To test this hypothesis we cultured UPEC biofilms for 24 hours, at which time we added FN075 or DMSO (vehicle control) at previously reported concentrations [29]. Biofilms were allowed to grow in the presence of compound/vehicle for 24 hours prior to quantitation by crystal violet staining and imaging by MALDI IMS (S4 Fig). Consistent with previous observations [30], DMSO treatment increased biofilm levels and CsgA expression compared to untreated controls (S4 Fig). Though these experiments were carried out under atmospheric conditions, DMSO can serve as an alternative terminal electron acceptor for *E. coli* [31]. This ability of DMSO may be contributing to the observed increase in biomass, though additional studies are needed to dissect the basis of biofilm increase in response to DMSO treatment. Colorimetric quantitation of biofilm levels also revealed a significant reduction in biomass with FN075-treatment of biofilms (*p* = 0.0089), compared to the DMSO-treated controls (S4 Fig). Consistent with the difference in biofilm levels, average MALDI IMS spectra normalized to the total ion current (TIC) indicated a higher level of overall signal within the DMSO-treated samples (S4 Fig). To account for the differences in biofilm levels between non-treated 48 hour biofilms, DMSO-treated, and the FN075-treated samples, mMass [32] software was used to normalize the overall intensity of the average spectra of each sample to the most abundant ion in the analysis (*m/z* ~7,280). These normalization parameters revealed an apparent increase in detection of the ion species corresponding to CsgA (*m/z* 13,036) within the FN075-treated sample, despite the reduction in overall biofilm levels (S4 Fig). IMS ion images for CsgA also appeared to show an increase in detectable CsgA monomers within the liquid-exposed region of the biofilm (S4 Fig). This is consistent with our hypothesis that FN075 treatment of a pre-formed biofilm would lead to an increase in monomeric CsgA, which would be more readily ionized and thus detected. Having validated the identity and localization of CsgA and FimA, we next sought to understand the basis of the spatial segregation of type 1 pili within UPEC biofilms.

### The presence of oxygen induces the expression of type 1 pili in UPEC

**Fig 5:**
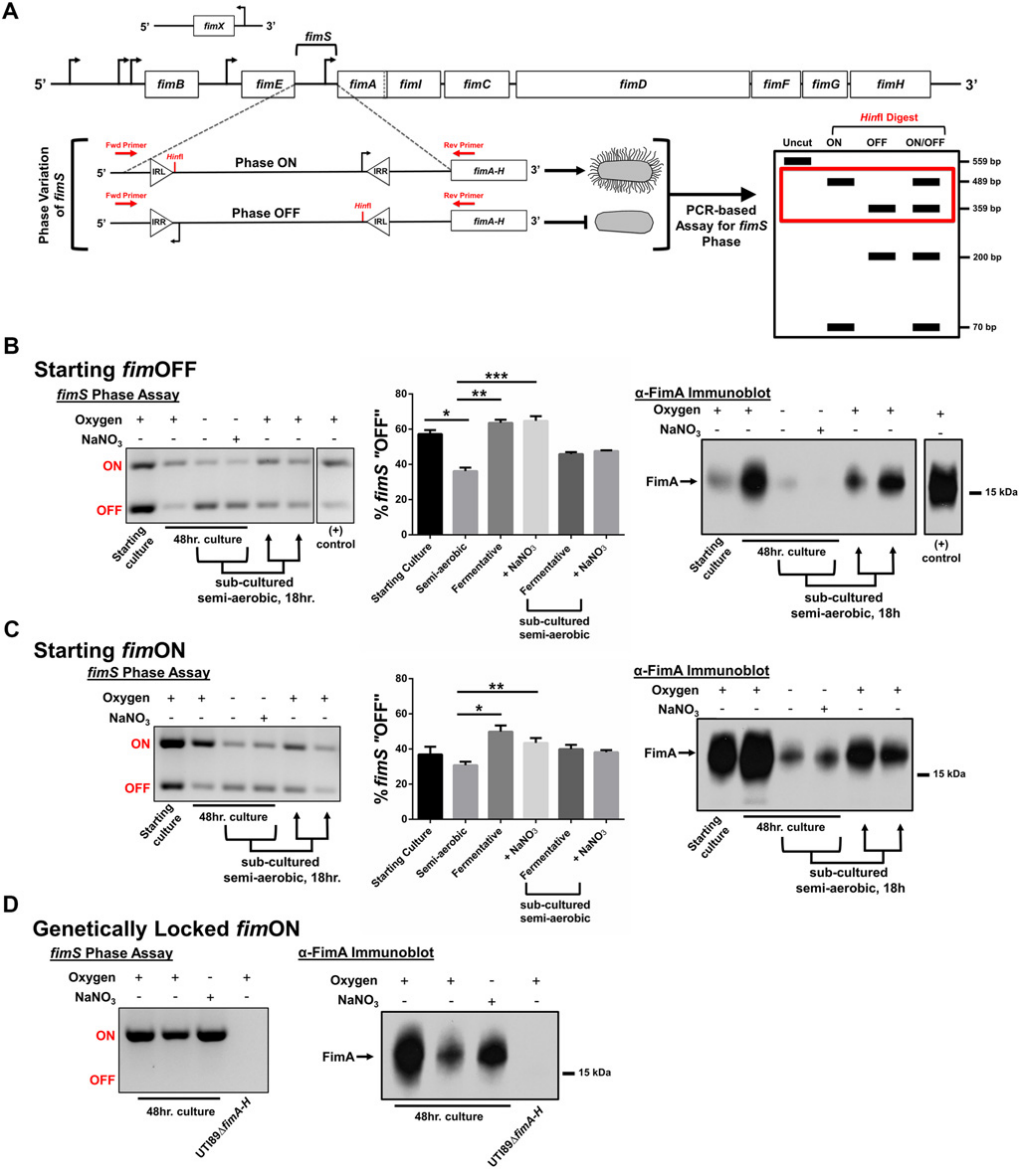
UPEC type 1 pili expression is repressed under oxygen-deplete growth conditions. **(A)** Schematic of the *fim* operon and the phase variable *fimS* promoter region. Promoter orientation is determined by PCR-based assay, followed by *Hinf*I digestion and analysis on 2% agarose gel. The red box in the schematic indicates the bands visualized in B-D. **(B)** Analysis with cultures starting primarily *fim*OFF cultured for 48 hours under semi-aerobic, fermentative, or anaerobic conditions in the presence of nitrate (NaNO_3_). The gel depicts a representative phase assay gel. The percentage *fim*OFF for each sample from multiple biological replicates is graphed as mean with SEM using GraphPad Prism 6 for each sample (starting culture/fermentative sub-culture/+NaNO_3_ subculture, n = 2; semi-aerobic/fermentative, n = 9; +NaNO_3_, n = 8). Statistical difference from semi-aerobic culture shown and determined by one-way ANOVA with Bonferroni’s multiple comparisons test using GraphPad (*p = 0.0019, ** and ***p = <0.0001). Statistically significant differences were also noted (but not indicated on the graph) between fermentative/fermentative sub-cultured [*p* = 0.0123], fermentative/+NaNO_3_ sub-cultured [*p* = 0.0316], +NaNO_3_/fermentative sub-cultured [*p* = 0.0072], and +NaNO_3_/+NaNO_3_ sub-cultured [*p* = 0.0185]. Corresponding anti-FimA immunoblots from the same samples used for each phase assay are shown. (C) Analyses as shown in B, for cultures starting predominantly *fim*ON. Phase assay quantitation taken from biological replicates of starting culture/fermentative sub-culture/+NaNO_3_ subculture, n = 2; semi-aerobic/fermentative/+NaNO_3_, n = 6. Statistical analysis performed as in B (*p = 0.0004, **p = 0.0344). Immunoblot is representative of five biological replicates. (D) Representative phase assay gel (n = 8) and anti-FimA immunoblot (n = 8) of cultures using UTI89_LON strain. Coomassie stained gels to verify equal loading and Ponceau S stained membranes to show equal transfer are provided in S6 Fig for the samples shown here.

The observation that type 1 pili-producing bacteria make up the top-most layer of the biofilm led us to the hypothesis that oxygen tension, at least in part, regulates the expression of type 1 pili. The *fim* gene cluster is under the control of a phase-variable promoter region (*fimS*), the orientation of which in UTI89 is directed by the action of site-specific recombinases FimB, FimE, and FimX (Fig. 5A) belonging to the lambda integrase family [33]. At least two other global transcriptional regulators, Lrp and IHF, have been proposed to bend the *fimS* DNA in order to bring the invertible repeats in close proximity to each other and allow for recombination [33,34]. We used a previously developed PCR-based “phase assay” [35] that can distinguish between the transcription-competent ON (*fim*ON) and transcription-incompetent OFF (*fim*OFF) orientations of the *fim* promoter (Fig. 5A), along with immunoblot analysis and transmission electron microscopy to evaluate whether oxygen is requisite for *fim* expression.

UTI89 was grown statically in either the presence or absence of oxygen in two different growth media (YESCA and Luria Bertani (LB)) and in two different temperature conditions (room temperature and 37°C) to evaluate the possibility that Fim localization to the air-exposed region was due to a nutritional or a temperature cue (S1 Table). Static growth at 37°C in LB media under atmospheric conditions enhances expression of UPEC type 1 pili [36–38]; these conditions were used as a positive control. UTI89Δ*fimA-H* was used as a negative control. Given the static nature of all culture methods, cultures grown in the presence of oxygen were termed “semi-aerobic”.

When starting these experiments from UPEC cultures that were primarily *fim*OFF, we observed that sub-culturing statically in the presence of oxygen induced expression of type 1 pili (Fig. 5B—“semi-aerobic”, S5 Fig, S6 Fig). However, regardless of growth medium or temperature, the *fim* promoter remained in the *fim*OFF orientation when bacteria were cultured in the absence of oxygen (fermentative conditions) (Fig. 5B and S5 Fig). When oxygen is not present, *E. coli* can utilize alternative terminal electron acceptors, such as nitrate, DMSO, TMAO, or fumarate [31]. Given that nitrate is the preferred alternative electron acceptor for *E. coli*, we assayed how anaerobic growth in the presence of nitrate (in the form of 40 mM sodium nitrate, NaNO_3_) would impact expression of type 1 pili. We observed that static cultures started *fim*OFF remained largely *fim*OFF during anaerobic growth in the presence of NaNO_3_ similar to what was observed with cultures grown fermentatively (Fig. 5B). When populations grown fermentatively or anaerobically with nitrate were sub-cultured into semi-aerobic conditions for 18 hours, the phase-variable promoter returned predominantly to the *fim*ON orientation, leading to increased FimA protein levels (Fig. 5B). These results suggested that the phase-switch from *fim*OFF to *fim*ON is affected by the bacterial respiration state, favoring aerobic respiration.

### Cell populations starting *fim*ON show an active switch to the *fim*OFF orientation under oxygen-deplete conditions

Previous studies indicated that multiple static sub-cultures under aerobic conditions enhance expression of type 1 pili by enriching for UPEC populations in which the *fim* promoter is *fim*ON [36,37]. We thus repeated our experiments starting from cultures that were pre-enriched for *fim*ON populations to test whether this would influence piliation in the absence of oxygen. Phase assays, FimA western blot analyses, and transmission electron microscopy (TEM) revealed that under fermentative conditions, the promoter actively inverted to the *fim*OFF orientation (Fig. 5C), leading to significantly fewer pili on the cell surface (Fig. 5C and S7 Fig). These data suggest that under fermentative conditions the phase-switch is preferably in the *fim*OFF orientation. Interestingly, growth of *fim*ON cells in the presence of nitrate partially preserved the *fim*ON state and production of type 1 pili on the surface (Fig. 5C and S7 Fig). The partial preservation observed under anaerobic growth in the presence of nitrate for populations starting *fim*ON suggests that anaerobic respiration does not impact the *fim*ON to *fim*OFF phase-switch. Together, these data suggest a regulatory mechanism that actively senses and responds to environmental oxygen levels, and/or bacterial respiration state, to control the expression of type 1 pili in UPEC by altering *fimS* promoter orientation.

### Type 1 pili expression is suppressed under oxygen-deplete conditions for cell populations genetically locked *fim*ON

In previous studies we created a UPEC strain (UTI89_LON) in which the *fim* promoter element is genetically locked into the transcription-competent *fim*ON orientation [38]. We postulated that if oxygen/respiration state only impacts the phase-state of the *fim* promoter, then UTI89_LON would be piliated when cultured in the absence of oxygen. When cultured under fermentative conditions, UTI89_LON exhibited a marked reduction in type 1 pili production, similar to wild-type (WT) UTI89, despite the “locked on” position of the promoter (Fig. 5D and S7 Fig). The phase state of the *fim* promoter in UTI89_LON was verified by phase assays (Fig. 5D) to exclude the possibility of mutations affecting the phase state under the conditions tested. These data point towards an additional regulatory mechanism that influences production of type 1 pili in a manner that is independent of the *fim* promoter switch.

Interestingly, anaerobic growth in the presence of nitrate induced *fim* gene expression in UTI89_LON (Fig. 5D), similar to the *fim*ON population shown in Fig. 5C (Fig. 5C-D and S7 Fig). Taken together, these observations suggest that the absence of oxygen impacts the phase state of the *fim* promoter element, and demonstrate that if the promoter is found in the *fim*ON orientation, the presence of an alternative electron acceptor is sufficient to induce transcription.

### S pili are expressed during fermentative growth conditions

**Fig 6:**
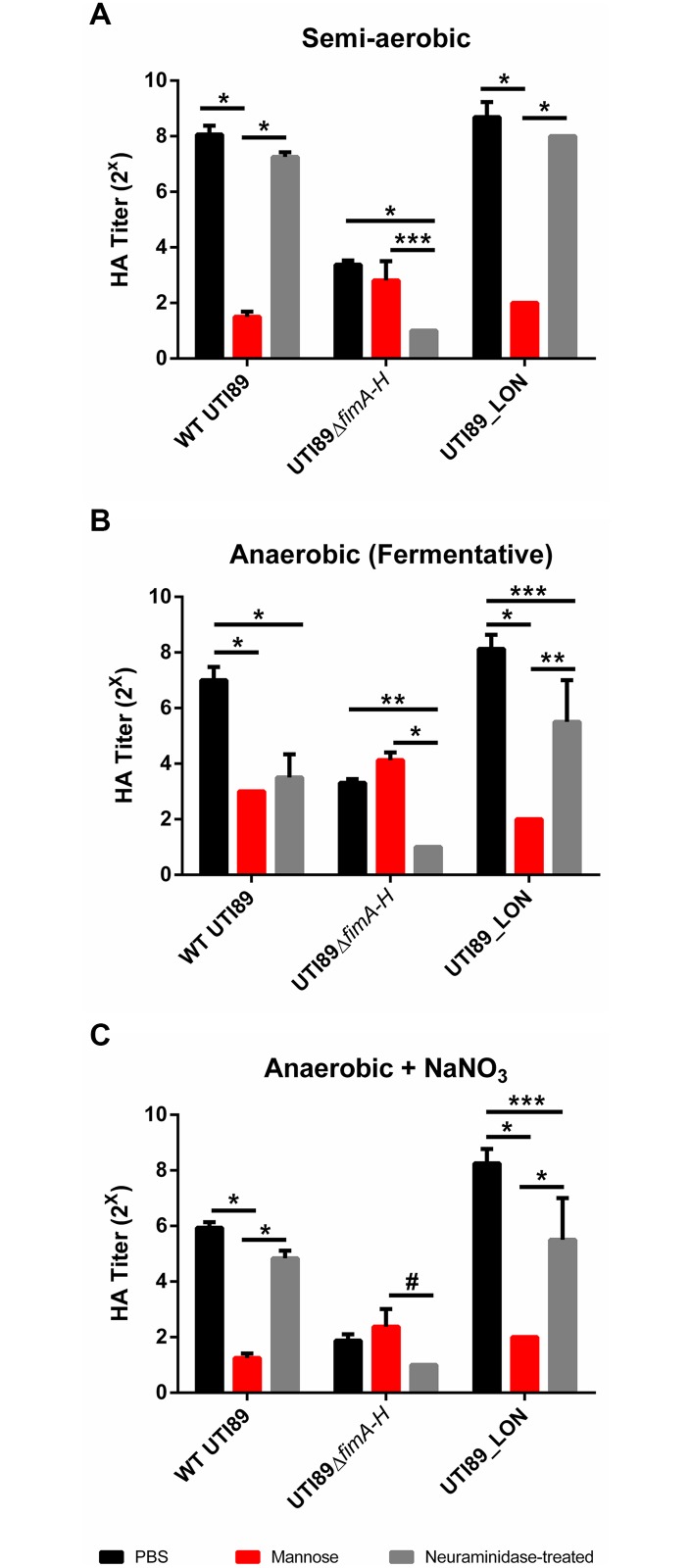
Suppression of type 1 pili under oxygen-deplete conditions enhances expression of S pili. Hemagglutination assay measuring type 1 (mannose-dependent) and S pili (sialic acid dependent) mediated agglutination under (A) semi-aerobic, (B) fermentative, and (C) anaerobic + NaNO_3_ growth conditions from cultures started from a population primarily *fim*ON. Data presented as mean with SEM, obtained from two technical replicates of two biological replicates. Statistical analysis performed as a two-way ANOVA with Bonferroni’s multiple comparisons test using GraphPad (^#^ *p* = <0.05, ***p = <0.01, *p = <0.001, *p = <0.0001).

Previous studies indicated that reduction in the expression of type 1 pili induces the expression of S pili under type 1 pili-inducing conditions [39–41]. We therefore evaluated the presence of S pili on the surface of the cell. Type 1 pili are characterized by their ability to bind mannosylated moieties [42]. An assay to evaluate the extent of type 1 pili in a UPEC population involves the agglutination of guinea pig red blood cells in the presence and absence of mannose. In bacteria that solely express type 1 pili, hemagglutination can be abolished by the addition of mannose to the agglutination reaction [42]. S pili bind sialic acid residues; therefore desialylation of red blood cells using neuraminidase prior to the agglutination assay abrogates S pili-dependent hemagglutination [43,44]. We combined these two approaches to establish the identity of the pili produced by UTI89 under anaerobic growth with cultures started from populations primarily *fim*ON. As expected, when WT UTI89 was grown statically in the presence of oxygen, hemagglutination (HA) was abolished in the presence of mannose and was unaffected by neuraminidase treatment (Fig. 6A), suggesting high numbers of type 1 pili. However, WT UTI89 grown under fermentative conditions exhibited lower HA titers that were inhibited by both mannose and by neuraminidase treatment (Fig. 6B), indicating that the observed agglutination was mediated by both type 1 and S pili. Given the inverse relationship between these two chaperone usher pathway (CUP) pili systems, the observable increase in S pili-mediated agglutination under fermentative growth conditions is an orthologous approach to demonstrate the down-regulation of type 1 pili in response to the lack of oxygen.

WT UTI89 grown anaerobically in the presence of nitrate exhibited overall lower HA titers compared to semi-aerobic and fermentative conditions (Fig. 6C). However, this agglutination was inhibited by mannose and was not significantly impaired by neuraminidase treatment, confirming the de-repression of type 1 pili expression by addition of nitrate and the subsequent down-regulation of S pili. UTI89_LON exhibited an HA profile that was similar to WT UTI89, suggesting that when the *fim* promoter is genetically locked in the *fim*ON orientation, it exerts a negative effect thereby repressing S pili expression (Fig. 6). UTI89Δ*fimA-H* yielded low HA titers under the three growth conditions tested and agglutination was not inhibited by mannose but was abolished when treated with neuraminidase, verifying that pili observed by TEM with the UTI89Δ*fimA-H* mutant are S pili (Fig. 6 and S7 Fig). These data demonstrate that the inverse relationship previously reported for type 1 and S pili [41] is maintained during growth in the absence of oxygen and that depletion of oxygen does not repress expression of all CUP pili systems.

## Discussion

This work shows MALDI IMS to be a strong analytical technology to study the spatial proteome of intact bacterial biofilms. Using a surface-associated biofilm setup that allowed for the formation of a biomass spanning two environmental niches (liquid versus air), we show that this imaging technology can be applied towards the interrogation of biofilm heterogeneity without *a priori* knowledge of protein targets of interest. Various mass spectrometric techniques have previously been applied for the study of microbial systems [45]. Laser desorption post-ionization mass spectrometry has been applied to analyze peptides involved in sporulation and bacterial competence [46], and secondary ion mass spectrometry (SIMS) was successfully used to analyze peptides involved in bacterial swarming [47]. MALDI IMS has been used successfully for the analysis of small molecules and metabolites within bacterial communities [48–51]. To date, only one other study has utilized MALDI IMS for the direct analysis of protein species within a bacterial community [52]. M.T. *et al*. used MALDI IMS for the analysis of peptides and proteins found at the site of interaction between *E. coli* and *Enterococcus faecalis* biofilms co-cultured on an agar surface, as well as within each individual biofilm [52]. Other than this initial study, little has been done to define the stratification of proteins within intact biofilms by IMS. Therefore, the application of MALDI IMS for the analysis of the intact spatial proteome of a single-species bacterial community represents an emerging approach that has the potential to offer new insights into the role and regulation of protein stratification within biofilms.

One caveat to MALDI-TOF IMS analyses of intact protein localization is that the species observed are typically limited to those most abundant within the sample or those that crystallize and ionize best with the MALDI matrix selected [25,53,54]. This limitation can restrict the sensitivity and dynamic range of the analytes observed by IMS. In turn, large molecular weight proteins or large polymeric protein complexes vital to biofilm formation, which are harder to ionize by MALDI and detect by time-of-flight mass analysis could be intrinsically excluded from the data. This caveat is exemplified by our curli fiber studies, where FN075 treatment increased the amount of detectable CsgA. Thus, orthologous approaches are still critical for validating MALDI IMS findings.

The profile of protein species observed can be expanded by varying the UV-absorbing matrix used for the analysis and by extending the overall *m/z* ion range analyzed (i.e. from 2,000–25,000 *m/z* to 2,000–40,000 *m/z*, and so on) [55]. The sensitivity of MALDI IMS can be refined further by increasing the spatial resolution at which the biofilm is imaged from the current resolution of 150 μm to as low as 20μm in order to better define stratification of subpopulations. We are currently developing both methods to enhance the number and type of protein species that can be localized within a single biofilm. While our approach clearly did not capture the global biofilm proteome, it simultaneously detected the spatial localization of up to 60 protein species within a single analysis; this represents a significant advancement compared to more traditional antibody- or fluorescent tag-based approaches that have been largely limited in the number of protein species visualized per analysis. In addition, the localization of proteins such as FimA and CsgA, which have been shown to play a crucial role in UPEC biofilm formation and pathogenesis but cannot be epitope-tagged due to their incorporation in macromolecular structures, also highlights the strength of this application.

MALDI IMS analyses revealed that type 1 pili-producing bacteria stratify above curli fiber-producing bacteria within the UPEC glass slide surface-associated biofilms interrogated in our studies (Fig. 3B). Similar UPEC biofilms have been previously shown to consist of an extracellular matrix comprised of curli and cellulose [7,28], with type 1 pili playing an accessory role in biofilm tensile strength [7]. The study by Hung *et al*., revealed that the bacteria on the air-exposed layer of a floating pellicle biofilm (formed during growth in the same media used in our studies), are morphologically distinct from those at the liquid interface [7]. In the same study, they also reported that disruption of *fim*-mediated adhesion did not ablate biofilm formation, but rather impaired biofilm integrity through the formation of large holes on the air-exposed side of the biomass [7]. Here, MALDI IMS demonstrated that type 1 pili are produced by the bacteria forming the topmost, air-exposed layer of the biofilm. In our studies, we observed that a pellicle biofilm typically surrounded the UPEC slides cultured for IMS analysis within 72–96 hours of starting the culture. If the slide-associated biofilm analyzed by MALDI IMS, is representative of a cross-section of the growing pellicle biomass biofilm, stratification of type 1 pili observed in surface-associated biofilms by IMS could help to explain the loss in tensile strength upon disruption of *fim*-mediated adhesion observed by Hung *et al*. However, it is important to note that the type of surface to which the bacteria adhere and the nutrient or surrounding environmental conditions can alter the genetic expression profiles within the biofilm community. Therefore, we recognize that the conclusions drawn here are representative of biofilms formed on a glass surface in a laboratory setting and may bear differences from cross-sections obtained from floating pellicles.

Bacterial biofilms constitute a serious problem in the healthcare setting. The unique heterogeneous architecture of the biofilm, combined with the composition of a self-secreted extracellular matrix, greatly hampers the penetrance and efficacy of bactericidal drugs and limits treatment options in the case of biofilm-related infection [21]. It is thus imperative to identify new strategies to combat or re-program how bacteria form these multicellular structures. Numerous studies identified the presence of bacterial subpopulations within bacterial biofilms and identified that these subpopulations execute unique “tasks” [56,57]. For example, in the benign *B. subtilis* biofilms, specific subpopulations produce extracellular matrix while others undergo sporulation [57,58]. Further studies indicated that *B. subtilis* biofilms are coated with a hydrophobin that renders the biofilm colony impervious to penetration [58]. In *E. coli* and other pathogens, metabolically inactive “persister” cells within the biofilm re-seed the infection upon cessation of antibiotic treatment [8,56,59]. Identifying the spatial proteome of biofilms may uncover markers for distinct subpopulations, thereby aiding in the development of new strategies for thwarting biofilm formation.

Our analyses so far revealed that induction of type 1 pili expression likely occurs on the topmost layer of the imaged biofilm due to the increased oxygen levels in this region. Previous studies reported that UPEC strains rely on the TCA cycle during infection [39,60] and that TCA cycle perturbations lead to a repression of *fim* gene expression and abrogation of intracellular bacterial community formation [39]. The studies described here show that there are at least two regulatory mechanisms that control expression of type 1 pili in the absence of oxygen; one that exerts its regulatory effect by influencing the *fim* promoter switch and another that acts independently of the *fim* promoter switch. Both of these mechanisms are engaged under fermentative growth, strongly suggesting that loss of the ability to use the electron transport processes imposes an energetic cost to the bacteria and necessitates the down-regulation of energetically expensive structures.

In probing the basis of these mechanisms, we have found that under fermentative conditions, there is no significant change in steady-state mRNA transcripts of the two main *fim* recombinases FimB and FimE (S8 Fig). We have also ruled out the involvement of the Anaerobic Respiration Control (Arc) two-component system (S8 Fig). It is likely that the effects on the phase-state of the *fim* promoter result from effects on the function of FimB and/or FimE as previously described [61]. Muller *et al*. elegantly demonstrated that CRP impacts *fim* gene expression by interfering with FimB function and repressing the expression of Lrp [61]. Other studies indicated that mutants deleted for the global regulator FNR had increased levels of Lrp under anaerobic growth conditions, suggestive of FNR down-regulating *lrp* expression in the absence of oxygen [62,63]. In the UPEC strain CFT073, Barbieri *et al*. have demonstrated that deletion of FNR suppresses expression of the FimB recombinase under atmospheric conditions [63]. We are currently investigating the involvement of FNR on modulating *fim* promoter switching in UPEC strain UTI89.

Use of alternative electron acceptors affords *E. coli* the ability to continue the electron transport processes under a variety of growth conditions, extending the range of environmental conditions they can withstand. Here we show that while incorporation of an alternative terminal electron acceptor (nitrate) partially preserved piliation in cells that had the promoter *fim*ON, it was unable to restore production of type 1 pili in cells with the promoter in the *fim*OFF orientation. We have attributed this effect to the ability of nitrate to serve as an alternative terminal electron acceptor. However, it is important to note that nitrate itself, as well as byproducts of nitrate respiration, specifically nitric oxide (NO), can also serve as a signaling molecule within the biofilm community [64–66]. NO has also been shown to have anti-biofilm abilities, suggesting possible role within biofilm signaling and maintenance [67]. We are currently in the process of confirming our results and examining the impact of the other preferred alternative terminal electron acceptors of *E. coli* (DMSO, TMAO, and fumarate), on type 1 pili expression under oxygen-deplete conditions.

Overall, the results of our nitrate studies are in agreement with our previous studies, in which a non-functional TCA cycle threw the *fim* switch in the *fim*OFF orientation [39]. Pathogenic extra-intestinal *E. coli* strains, such as UPEC, typically thrive in the gastrointestinal tract of humans and other warm-blooded animals where oxygen is limited. As UPEC exit the gut and ascend the urethra to eventually colonize the urinary tract, they undergo multiple metabolic transitions between aerobic and anaerobic growth states. Each of these transitions is accompanied by fluctuations in oxygen tension from strictly anaerobic to highly oxygenated, to semi-aerobic. The bacterial cells respond to these fluctuations by modulating central metabolic pathways for carbon and energy flow, which in turn impact expression of a battery of targets including virulence factors. Together with previous reports [39,60], the studies described here corroborate a direct link between respiration state and the expression of adhesive fibers that has multiple regulatory checkpoints, possibly to account for the diverse fluctuations in oxygen tension encountered by UPEC. Our study also suggests that oxygen gradients determine fiber stratification within the biofilm, which may contribute to overall integrity.

Collectively, our studies used MALDI IMS to begin to define the spatial stratification of distinct bacterial subpopulations within UPEC biofilms based on differential protein expression profiles. Extrapolating from observations made by MALDI IMS, we discovered that type 1 pili-producing bacteria constitute the uppermost layer of UPEC biofilms under the conditions tested, and we identified two new UPEC regulatory mechanisms that control the expression of type 1 pili in response to oxygen and/or bacterial respiration state. These findings highlight how MALDI IMS can drive the identification and characterization of biofilm subpopulations, leading to a greater understanding of their role and regulation within the biofilm.

## Materials and Methods

### Bacterial strains

For these studies we used the UPEC cystitis isolate UTI89 [24]. Previously constructed UTI89 mutants used in this study are UTI89Δ*fimA-H* (gift from Dr. Scott Hultgren); UTI89_LON [38]; and UTI89Δ*arcA* (gift from Dr. Matthew Chapman). UTI89Δ*hupA* and UTI89Δ*yahO* were created using the previously established λ Red recombinase methods [68] and the following primers (Integrated DNA Technologies): *hupA*_Fwd (5’–TTACTTAACTGCGTCTTTCAGTGCCTTGCCAGAAACAAATGCCGGTACGTGTGTAGGCTGGAGCTGCTT–3’) / *hupA*_Rev (5’-ATGAACAAGACTCAACTGATTGATGTAATTGCAGAGAAAGCAGAACTGTCCATATGAATATCCTCCTTAG-3’); *yahO*_Fwd (5’-ATGAAAATAATCTCTAAAATGTTAGTCGGTGCGTTAGCGTTTGCCGTTACGTGTAGGCTGGAGCTGCTTC-3’) / *yahO_Rev* (5’-TTACTTCTTCTTATAAATATTTGCCGTGCCGTGAATCTTATTGTCAGTTTCATATGAATATCCTCCTTAG-3’).

### Biofilm growth conditions

All strains were grown overnight in Lysogeny broth (LB) (Fisher), pH 7.4, at 37°C with shaking, unless otherwise specified. Overnight cultures were then sub-cultured in 1.2x Yeast-Extract/Casamino Acids (YESCA) broth [43]. Bacterial suspensions were then dispensed in 50 mL conical tubes containing ITO-coated glass slides (Delta Technologies) and cultured for 48 hours at room temperature. After culture, slides were removed, rinsed with water to remove non-adherent bacteria and stored at -80°C until analysis.

### Biofilm quantitation

Biofilms were quantified as previously described [43]. Crystal violet stained biofilms were removed from ITO slides using 35% acetic acid and transferred to 96-well plates for absorbance readings. Absorbance at 570 nm was determined using a BioRad Model 680 microplate reader (BioRad). Data are presented as the average absorbance from at least three independent experiments. Statistical analysis was performed using a two-tailed unpaired Student’s *t*-test (GraphPad Prism 6).

### Microscopy

**Scanning electron microscopy (SEM)**. Bacterial biofilms grown as described for MALDI IMS were treated for SEM as previously described [69]. Samples were dried at the critical point, mounted onto aluminum sample stubs and sputter coated with gold-palladium. A small strip of silver paint was applied to the sample edge, and biofilms were imaged with an FEI Quanta 250 Field-emission gun scanning electron microscope (FEI). At least two biological replicates were imaged for each sample preparation and representative images were collected.

**Transmission electron microscopy (TEM)**. TEM analyses were performed as outlined previously [40]. Briefly, 100 μL of normalized bacterial cultures (OD_600_ = 1.0) from each condition were centrifuged at 4,000 rpm for 10 minutes and resuspended in 50 μL of TEM fixative (2.5% glutaraldehyde in 100mM sodium cacodylate (Electron Microscopy Sciences)) for 1 hour at room temperature. Samples were then deposited onto glow-discharged formvar-/carbon-coated copper grids (Electron Microscopy Sciences) for 60 seconds and stained with 1% uranyl acetate for 90 seconds. Samples were then analyzed on a Phillips/FEI T-12 Transmission Electron Microscope (FEI).

**Immuno-fluorescence by Super-resolution Structured Illumination Microscopy (SIM)**. The α-CsgA antibody was provided by Dr. Matthew Chapman at the University of Michigan. UPEC biofilms were grown for 48 hours as previously described. Biofilms were fixed in 4% paraformaldehyde in phosphate-buffered saline (PBS) for 30 minutes at room temperature and blocked in 5% BSA overnight at 4°C. Biofilms were immuno-stained with α-CsgA (1:1000) for 1 hour at room temperature, followed by 3 washes in PBS and secondary detection with Alexa Fluor-555 goat anti-rabbit (1:1000) (Life Technologies) for 1 hour at room temperature. Samples were washed 3 times in PBS and mounted under a 1.5 size coverslip (Fisher Scientific) using ProLong Gold antifade reagent containing DAPI for DNA counterstain (Life Technologies). Cells were imaged using a GE/Applied Precision DeltaVision OMX in SIM mode with 1.516 immersion oil at 63X magnification. Post-data acquisition processing was performed using SoftWorx for OMX. Images were processed for contrast enhancement and cropping in Photoshop. With the exception of x-y sections (z stacks), images are shown as maximum intensity projections through the entire imaged area (ranging from 3–6 μm in z, 40 μm in x-y). Videos depicting three-dimensional reconstruction of biofilms were generated using the Volume Viewer in Progressive mode in SoftWorx for OMX.

### Optical profilometry

Surface analysis was performed on crystal violet stained biofilms using a Zeta-20 True Color 3D Optical Profilometer (Zeta Instruments) at 20x magnification. Fifty microns were z-stacked to create the profiles at 0.2 microns/step. Images were reconstructed using a 10% optical overlap in stitching. Optical images of crystal violet stained biofilms were obtained using a Leica SCN400 Digital Slide Scanner (Leica Microsystems) at 20x magnification in manual bright field mode.

### Matrix-Assisted Laser Desorption/Ionization (MALDI)—Time-of-flight (TOF) Imaging Mass Spectrometry (IMS)

Biofilms grown on ITO-coated glass slides were washed to remove interfering salts and lipids in sequential 30-second washes of 70, 90, and 95% HPLC-grade ethanol (Fisher Scientific). Matrix comprising 15 mg/mL 2,5-dihydroxybenzoic acid (DHB) (Fisher Scientific) and 5 mg/mL α-Cyano-4-hydroxycinnamic acid (CHCA) (Sigma-Aldrich) was applied using a TM-Sprayer (HTX Imaging), and samples were vapor rehydrated with 10% acetic acid. Samples were analyzed using a Bruker Autoflex Speed mass spectrometer (Bruker Daltonics) in linear positive ion mode. Each pixel contains an average of 200 spectra. Images were collected at 150 micron (μm) lateral resolution. Data were analyzed using FlexImaging 3.0 Build 42 (Bruker Daltonics). Datasets were normalized to total ion current unless otherwise indicated. Ion intensity maps were extracted for each range of interest and were plotted using the maximum intensity within the range. (Detailed MALDI-TOF IMS methods are found in S1 Methods).

### Protein fractionation and identification

To identify 48-hour UPEC biofilm *m/z* ion species observed by IMS, multiple slide-associated biofilms were lysed and pooled together. Lysates were sonicated, centrifuged, and supernatants dried by vacuum centrifugation (Thermo Scientific). Samples were resuspended and fractionated using C8 (Grace Vydac) or C18 (Phenomenex) reversed-phase high performance liquid chromatography (HPLC) (Waters). Fractions were analyzed for *m/z* ions corresponding to those observed in the IMS analyses, subjected to in-solution tryptic digestion, and submitted to the Vanderbilt University Mass Spectrometry Research Center Proteomics Core for LC-MS/MS identification (Detailed methods in S1 Methods). For validation of FimA protein identification, 48-hour biofilms of the UTI89Δ*fimA-H* were cultured as described above and analyzed by MALDI IMS. For validation of HupA and YahO protein identifications, 48-hour static liquid cultures (in 1.2x YESCA) of UTI89Δ*hupA* and UTI89Δ*yahO* were grown. After 48 hours, an aliquot of liquid culture was removed and pelleted. Pellets were then lysed with a volume of 35% acetic acid, and centrifuged to pellet debris. Lysates were then analyzed by MALDI-TOF MS (Bruker Daltonics) using the same matrix and parameters for IMS analyses.

### FN075 experiments

FN075 was prepared and characterized as described previously [29,70]. UPEC biofilms were cultured as described above for 24 hours. After 24 hours the preformed biofilm was treated with either 125 μM FN075 dissolved in 100% dimethyl sulfoxide (DMSO), an equivalent volume of 100% DMSO (vehicle control), or an equivalent volume of fresh YESCA media (negative control) and allowed to develop for another 24 hours. Slides were then removed and processed as described above. Biofilms were quantified and analyzed by MALDI IMS as described above.

### Growth conditions for analysis of oxygen-dependent *fim* expression in UPEC

WT UTI89 and mutant strains were cultured under media and growth conditions listed in Supplemental Table 1 (S1 Table). Cultures starting *fim*OFF were begun from overnight shaking cultures, and cultures starting *fim*ON were begun from overnight statically grown cultures, both in LB media at 37°C. Oxygen-deplete cultures were grown in an anaerobic chamber maintained at 0% oxygen with between 2–3% hydrogen. Alternative terminal electron acceptor samples were treated with 40mM sodium nitrate (NaNO_3_) (Sigma-Aldrich). All cultures were grown for 48 hours to mimic biofilm growth conditions used in IMS analyses. After 48 hours, cultures were normalized to an OD_600_ of 1.0 with sterile PBS for phase assay and immunoblot analysis.

### *fim* phase assays

Phase assays were performed as previously described [35] using 100 ng of genomic DNA, or an aliquot of normalized cells (OD_600_ 1.0) and with the following modifications: Primers Phase_L (5’-GAGAAGAAGCTTGATTTAACTAATTG-3’), and Phase_R (5’-AGAGCCGCTGTAGAACTCAGG-3’) were used and the PCR was performed using the following parameters: 95°C—5min, 30 cycles (95°C—45sec, 50°C—20sec, 72°C—45sec), 72°C—5min. To determine the proportion of the population *fim*ON vs. *fim*OFF, mean pixel intensity of the bands at 489 bp (*fim*ON) and 359 bp (*fim*OFF) was determined within each sample using Adobe Photoshop CS6 (Adobe Systems). Background taken from a blank area of the gel at a position equivalent to each band, was subtracted. The mean intensity of the *fim*ON and *fim*OFF band for each sample was then summed, and the percentage ON vs. OFF was then determined for each sample. The percentage of each sample *fim*OFF was then plotted with GraphPad Prism 6 (GraphPad Software Inc.), and statistical analysis was performed using a one-way ANOVA with Bonferroni’s multiple comparisons test.

### Immunoblot analysis

Immunoblots probing for FimA were performed as previously described [43]. Briefly, cultures were normalized to an OD_600_ = 1.0 and 1 ml of normalized cultures was pelleted by centrifugation. Normalized cell pellets were suspended 1x Laemmli sample buffer (BioRad) containing 5% 2-mercaptoetahnol (Sigma-Aldrich). Samples were acidified with 1M hydrochloric acid (HCl), heated at 100°C for 10 minutes, and then neutralized with 1N sodium hydroxide (NaOH). Samples were then resolved on a 16% SDS-PAGE gel. Following SDS-PAGE, proteins were transferred to nitrocellulose using the Trans-Blot Turbo Transfer System (BioRad), (7 minute transfer at 1.3A and 25V). Transfer efficiency was verified with Ponceau S (Sigma-Aldrich). Stains corresponding to blots shown in Fig. 5 are included in S6 Fig. Following transfer, membranes were blocked with 5% non-fat milk in 1x TBST overnight at 4°C. After blocking, membranes were washed 2x with 1x TBST and incubated with primary anti-FimA antibody [1:5,000] [43] for 1 hour at room temperature, washed 2x with 1x TBST, and incubated with HRP-conjugated goat—anti-rabbit secondary antibody (Promega) for 30 minutes at room temperature. Following secondary antibody application membranes were washed 3x with 1x TBST, treated with SuperSignal West Pico Chemiluminescent Substrate (Thermo Scientific), and bands visualized on x-ray film (MidSci). Immunoblots probing for CsgA were performed in a similar fashion with the exception that cell pellets were first solubilized in 100% formic acid, which was then evaporated prior to re-constitution in 1x SDS sample buffer, as previously described [29]. The anti-CsgA antibody was used at a 1:10,000 dilution.

### Hemagglutination assays

Hemagglutination assays were performed as described previously [43]. Guinea pig erythrocytes were obtained from the Colorado Serum Company. Erythrocyte de-sialylation was performed using *Clostridium perfringens* neuraminidase (New England BioLabs) for 2 hours at 37°C with gentle agitation.

### *fimB* and *fimE* qPCR analysis

RNA extraction, reverse transcription, and real-time quantitative PCR were performed as previously described [71]. qPCR analysis was performed with three concentrations of cDNA (50 ng, 25 ng, 12.5 ng) each in triplicate for each sample, and internal DNA gyrase (*gyrB*) levels were used for normalization. The following primers (Integrated DNA Technologies) were used for amplification; *fimB*_Fwd (5’—GCATGCTGAGAGCGAGTCGGTA—3’), *fimB*_Rev (5’—GGCGGTATACCAGACAGTATGACG—3’), *fimE*_Fwd (5’—ATGAGCGTGAAGCCGTGGAACG—3’), *fimE*_Rev (5’—TATCTGCACCACGCTCAGCCAG—3’), *gyrB*_L (5’—GATGCGCGTGAAGGCCTGAATG—3’), *gyrB*_R (5’—CACGGGCACGGGCAGCATC—3’). The following probes (Applied Biosystems) were used for quantitation; *fimB* (5’– 6FAM-TCATCCGCACATGTTAC-MGBNFQ—3’); *fimE* (5’—NED-CGGACCGACGCTATAT-MGBNFQ—3’); *gyrB* (5’—VIC-ACGAACTGCTGGCGGA-MGBNFQ—3’).

## Supporting Information

S1 Fig IMS sample preparation methods do not alter UPEC biofilm architecture. (A) Representative SEM micrographs of unwashed and ethanol-washed UPEC biofilms. Representative micrographs from at least two biological replicates are shown. Magnification shown, 10,000x; scale bar = 5 μm. (B) Biofilm quantitation by crystal violet staining. Graph depicts quantified biofilm of ethanol-washed and unwashed biofilms measured at 48 hours post-seeding. Data are presented as the mean with the standard deviation. EtOH, ethanol; *Statistical analysis was performed using two-tailed unpaired Student’s *t*-test (n = 9, *p* = 0.7864) (C) Optical profilometry showing areas of highest bacterial density on the conductive slide.
(TIF)

S2 Fig IMS ion overlay images reveals stratification of identified proteins within the UPEC biofilm. Representative IMS images depicting the localization and relative abundance of identified UPEC protein species relative to each other in the biofilm. Overlays are presented as a single color intensity map with representative intensity scale shown in white. The top-row heat map intensity (10–100%) indicates the relative abundance and localization of each protein species in the biofilm.
(TIF)

S3 Fig Mass spectrometry analysis of gene deletion mutants validates IMS ion identifications for FimA, HupA, and YahOUp. (A) IMS analysis of UTI89Δ*fimA-H* reveals loss of ion at *m/z* 16,269, corresponding to FimA. A representative single spectrum for the UTI89Δ*fimA-H* mutant (red) is shown, compared to an average spectrum taken from two biological replicates of wild-type (WT) UTI89 (black) after 48 hours of growth. (B–C) MALDI mass spectrometry analysis of lysed UTI89Δ*hupA* (B) and UTI89Δ*yahO* (C) bacteria pellets. (B) Traditional proteomics had identified the ion at *m/z* 9,535 as the transcriptional regulator, HupA (Table 1). Analysis of the UTI89Δ*hupA* mutant (green) indicates a loss of this ion peak. (C) Traditional proteomics had identified the ion at *m/z* 7,718 as the uncharacterized protein factor, YahO (Table 1). Analysis of the UTI89Δ*yahO* mutant (blue) indicates a loss of this ion peak. All spectra were imported to the mMass software, baseline subtracted, smoothed, and normalized to the most abundant ion in the spectra.
(TIF)

S4 Fig MALDI IMS detects biofilm responses to external stimuli. (A) Structure of the compound FN075 and biofilm quantitation for DMSO- and FN075-treated biofilms using crystal violet staining. Data presented as mean with the standard deviation (n = 2 for each condition). Statistical analysis was performed using a two-tailed unpaired Student’s *t*-test (*p = 0.0037, **p = 0.0089, ***p = 0.0325). (B) Immunoblot for CsgA protein levels between 48-hour cultures, and cultures treated with equivalent volumes of either YESCA media, DMSO (vehicle control), or 125 μm FN075 in DMSO after 24-hours and cultured for another 24-hours. Blot shown is representative of two technical replicates of two biological replicates. (C) Average raw and normalized MALDI IMS spectra from non-treated 48-hour (Green), DMSO-treated (Black), and FN075-treated (Red) biofilm IMS analyses. The average spectra are a result of two biological replicates for each condition after normalization to the total ion current using FlexImaging software. Average spectra were then imported to the mMass Software, background-subtracted and smoothed, and normalized to the most intense ion in the spectra at *m/z* 7,280. (D) IMS localization and abundance of CsgA within vehicle- or FN075-treated biofilms reveals an increase in distribution throughout the liquid-exposed region of the biofilm. Images shown for each condition are representative of two biological replicates and were processed as described in Fig. 2. Scale bar = 5 mm.
(TIF)

S5 Fig UPEC type 1 pili expression is repressed under oxygen-deplete growth conditions regardless of growth medium and temperature. (A) Phase assay and FimA immunoblot analysis of WT UTI89 cultured in 1.2x YESCA media at room temperature under semi-aerobic and fermentative growth conditions. Phase assay quantitation, n = 3. Statistical analysis performed by two-tailed unpaired Student’s *t*-Test in GraphPad Prism 6, with determined *p*-values shown. Immunoblot representative of n = 4 analyses. (B) Phase assay and FimA immunoblot analysis of WT UTI89 cultured in 1.2x YESCA media at 37°C under semi-aerobic and fermentative growth conditions. Phase assay quantitation, n = 1. Immunoblot representative of n = 2 analyses. (C) Phase assay and FimA immunoblot analysis of WT UTI89 cultured in LB media (pH 7.4) at room temperature under semi-aerobic and fermentative growth conditions. Phase assay quantitation, n = 1. Immunoblot representative of n = 2 analyses. Statistical analysis of phase quantitation not performed for (B) and (C) due to insufficient number of biological replicates. All data presented as outlined in Fig. 2. All cultures for the analyses in A-C were started from primary overnight cultures grown at 37°C with shaking conditions therefore each population began primarily phase *fim*OFF.
(TIF)

S6 Fig Equal loading and transfer verification of FimA immunoblots. (A) Coomassie stained gels post-transfer for the immunoblots presented in Fig. 5. (B) Ponceau S staining of membranes post-transfer for immunoblots shown in Fig. 5. Together these data show equal loading and protein levels of gels pre-transfer and equal transfer to the membranes used for immunoblotting.
(TIF)

S7 Fig TEM analysis reveals the extent of surface piliation in UPEC under semi-aerobic, fermentative, and anaerobic/nitrate growth conditions. Representative TEM micrographs for each strain under the three tested growth conditions. Cultures were initiated from static overnight stocks and cultured statically for 48 hours in LB media (pH 7.4) at 37°C under the respective growth condition. Scale bar = 500 nm.
(TIF)

S8 Fig Oxygen-dependent regulation does not affect FimB and FimE recombinase levels and is independent of the ArcAB two-component system. (A) qPCR analysis of *fimB* and *fimE* steady-state mRNA transcript levels from semi-aerobic and fermentative cultures started from WT UTI89 populations primarily *fim*OFF (shaking), WT UTI89 populations primarily *fim*ON (static), or genetically locked *fim*ON (UTI89_LON, started from shaking). RNA was extracted from bacterial pellets obtained from cultures presented in Fig. 5. Data is graphed as the mean ± SEM from two independent biological replicates, with three technical replicates of three different cDNA concentrations used for each biological replicate (9 technical reads per bio replicate). All data are normalized to the internal DNA gyrase (*gyrB*) values calculated within each sample and presented as the fold-change from WT UTI89 cultures started from primarily *fim*OFF populations grown under semi-aerobic conditions. Statistical analysis was performed via two-way ANOVA with Bonferroni’s multiple comparisons test. No statistical differences were noted. (B) Phase assay and FimA immunoblot with UTI89Δ*arcA* indicates this oxygen-dependent regulation of *fim* expression is not governed by the ArcAB two-component system. Data shown was obtained from cultures starting from populations primarily *fim*OFF (shaking) and grown in 1.2x YESCA media at room temperature. Phase assay quantitation, n = 3. Statistical analysis performed by two-tailed unpaired Student’s *t*-Test in GraphPad Prism 6, with determined *p*-values shown. Immunoblot representative of n = 5 analyses.
(TIF)

S1 Video Immuno-fluorescence by Super-resolution Structured Illumination Microscopy (SIM) of CsgA localization at the biofilm air-liquid interface. Video depicts a representative three-dimensional reconstruction of the biofilm at the air-liquid interface with DAPI staining for nucleic acids (Blue) and Alexa Fluor-555 conjugated secondary antibody detection of α-CsgA antibody (Red). Video was generated using the Volume Viewer in Progressive mode in SoftWorx for OMX. Image is representative of two biological replicates analyzed.
(MOV)

S1 Table Growth media and conditions used for analysis of type 1 pili expression in UPEC. Listed are the growth media, temperatures, and oxygen growth conditions under which we examined the expression of type 1 pili from the UPEC strain UTI89. All cultures were grown statically for 48 hours prior to analysis for pili expression. NaNO_3_, sodium nitrate.
(DOCX)

S1 Dataset Reproducible ion species observed by MALDI-TOF IMS. Ions presented were observed in at least 5 biological replicates, and their relative localization within the biofilm is described. The depicted mass-to-charge (*m/z*) ratio of each selected ion was determined after internal calibration of the total ion current-normalized average spectrum using mMass software. Internal calibration used the theoretical mass minus the signal peptide for proteins identified in the LC-MS/MS analyses to obtain the best mass accuracy for the data (as described for Fig. 2).
(XLSX)

S1 Methods Technical specifications and more in-depth methods for, MALDI-TOF IMS analyses of 48-hour UPEC biofilms and for proteomic methods for the identification of ion species observed by MALDI-TOF IMS. (DOCX)
