# Supplementary figures and images for: Adhesive Fiber Stratification in Uropathogenic Escherichia coli Biofilms Unveils Oxygen-Mediated Control of Type 1 Pili
Source: PLoS Pathog. 2015 Mar 4;11(3):e1004697. doi: 10.1371/journal.ppat.1004697 (PMC4349694; doi:10.1371/journal.ppat.1004697)

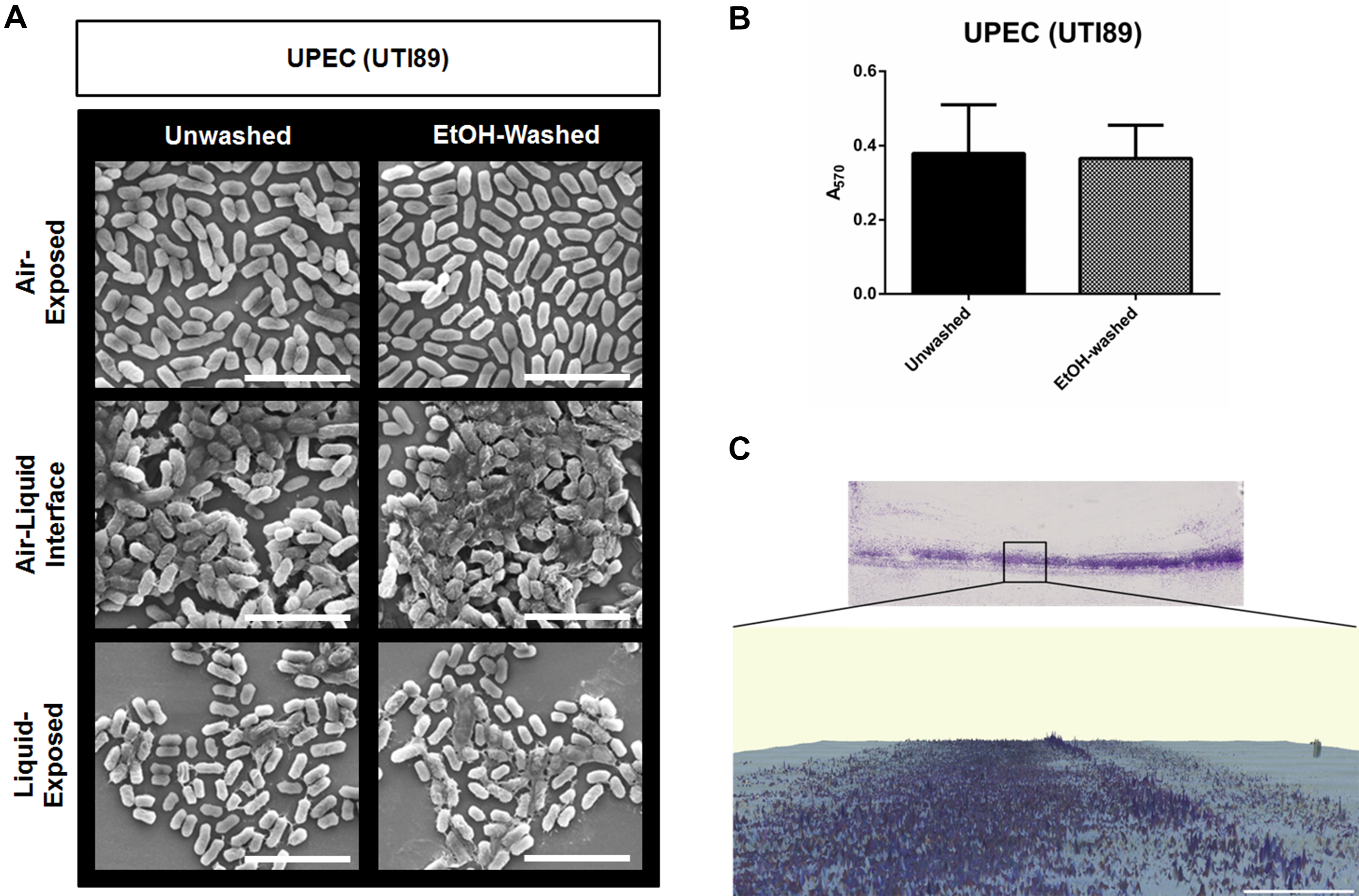

Supplement: S1 Fig — (A) Representative SEM micrographs of unwashed and ethanol-washed UPEC biofilms. Representative micrographs from at least two biological replicates are shown. Magnification shown, 10,000x; scale bar = 5 μm. (B) Biofilm quantitation by crystal violet staining. Graph depicts quantified biofilm of ethanol-washed and unwashed biofilms measured at 48 hours post-seeding. Data are presented as the mean with the standard deviation. EtOH, ethanol; *Statistical analysis was performed using two-tailed unpaired Student’s t-test (n = 9, p = 0.7864) (C) Optical profilometry showing areas of highest bacterial density on the conductive slide. (TIF) [file ppat.1004697.s001.tif]

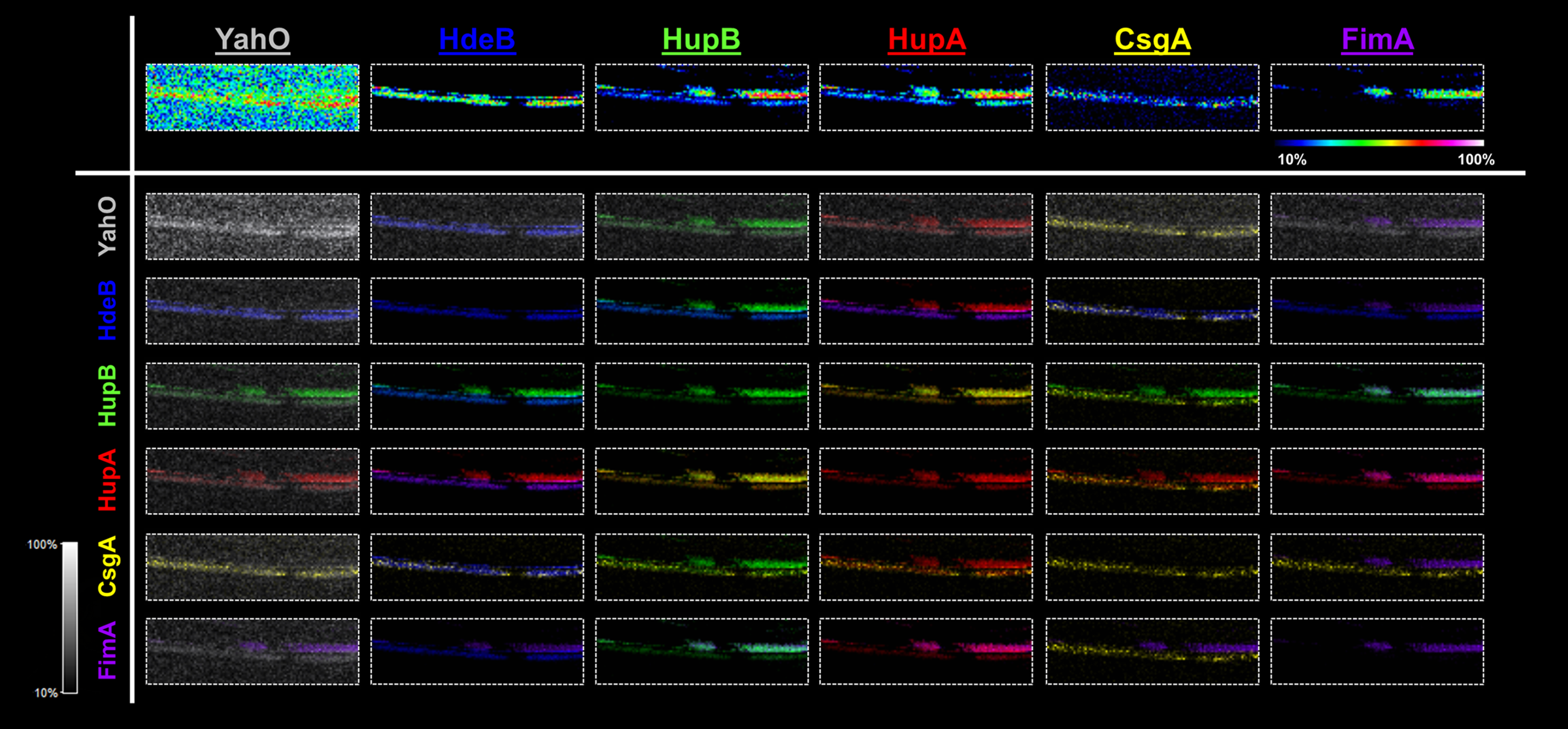

Supplement: S2 Fig — Representative IMS images depicting the localization and relative abundance of identified UPEC protein species relative to each other in the biofilm. Overlays are presented as a single color intensity map with representative intensity scale shown in white. The top-row heat map intensity (10–100%) indicates the relative abundance and localization of each protein species in the biofilm. (TIF) [file ppat.1004697.s002.tif]

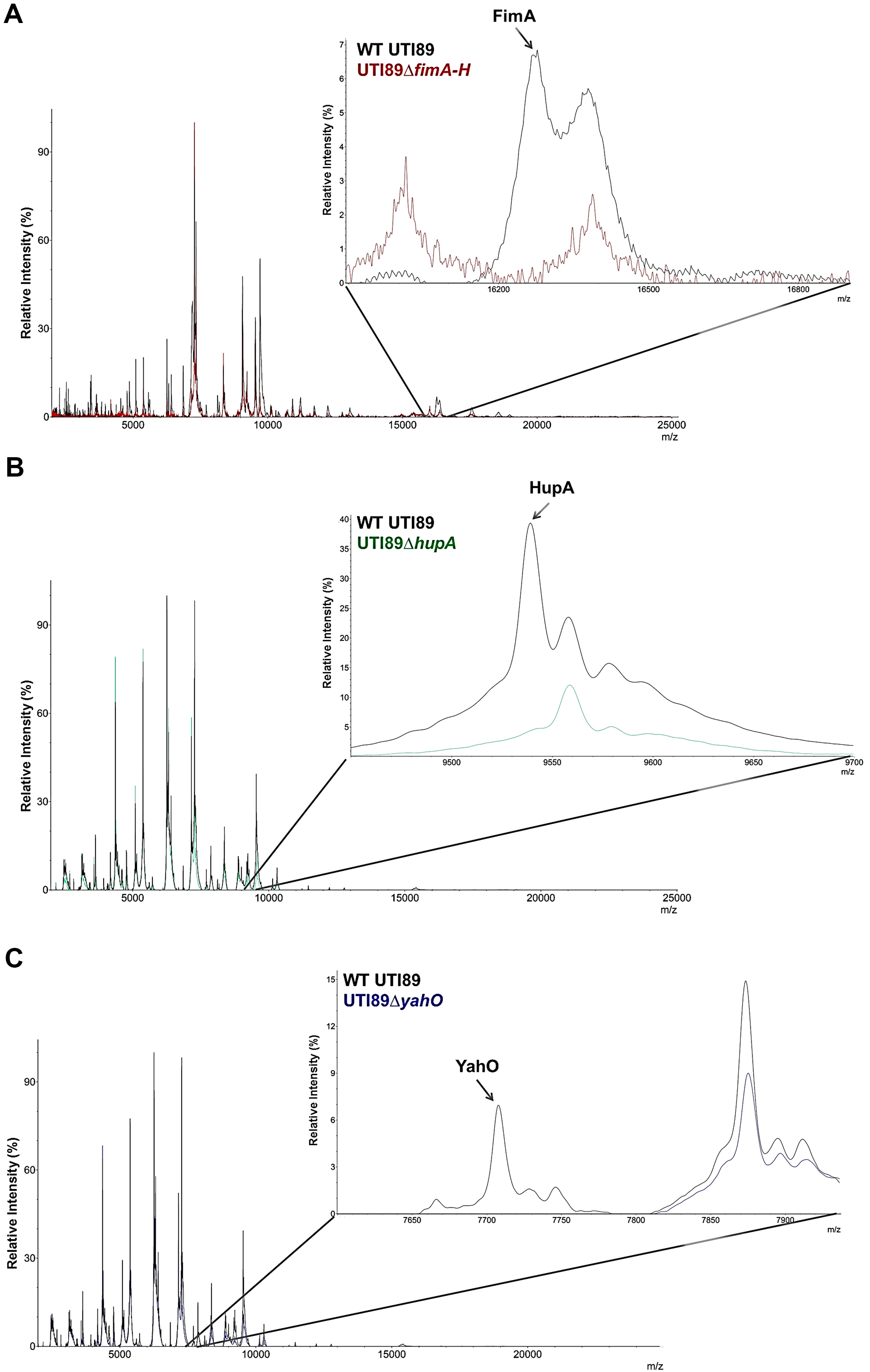

Supplement: S3 Fig — (A) IMS analysis of UTI89ΔfimA-H reveals loss of ion at m/z 16,269, corresponding to FimA. A representative single spectrum for the UTI89ΔfimA-H mutant (red) is shown, compared to an average spectrum taken from two biological replicates of wild-type (WT) UTI89 (black) after 48 hours of growth. (B–C) MALDI mass spectrometry analysis of lysed UTI89ΔhupA (B) and UTI89ΔyahO (C) bacteria pellets. (B) Traditional proteomics had identified the ion at m/z 9,535 as the transcriptional regulator, HupA (Table 1). Analysis of the UTI89ΔhupA mutant (green) indicates a loss of this ion peak. (C) Traditional proteomics had identified the ion at m/z 7,718 as the uncharacterized protein factor, YahO (Table 1). Analysis of the UTI89ΔyahO mutant (blue) indicates a loss of this ion peak. All spectra were imported to the mMass software, baseline subtracted, smoothed, and normalized to the most abundant ion in the spectra. (TIF) [file ppat.1004697.s003.tif]

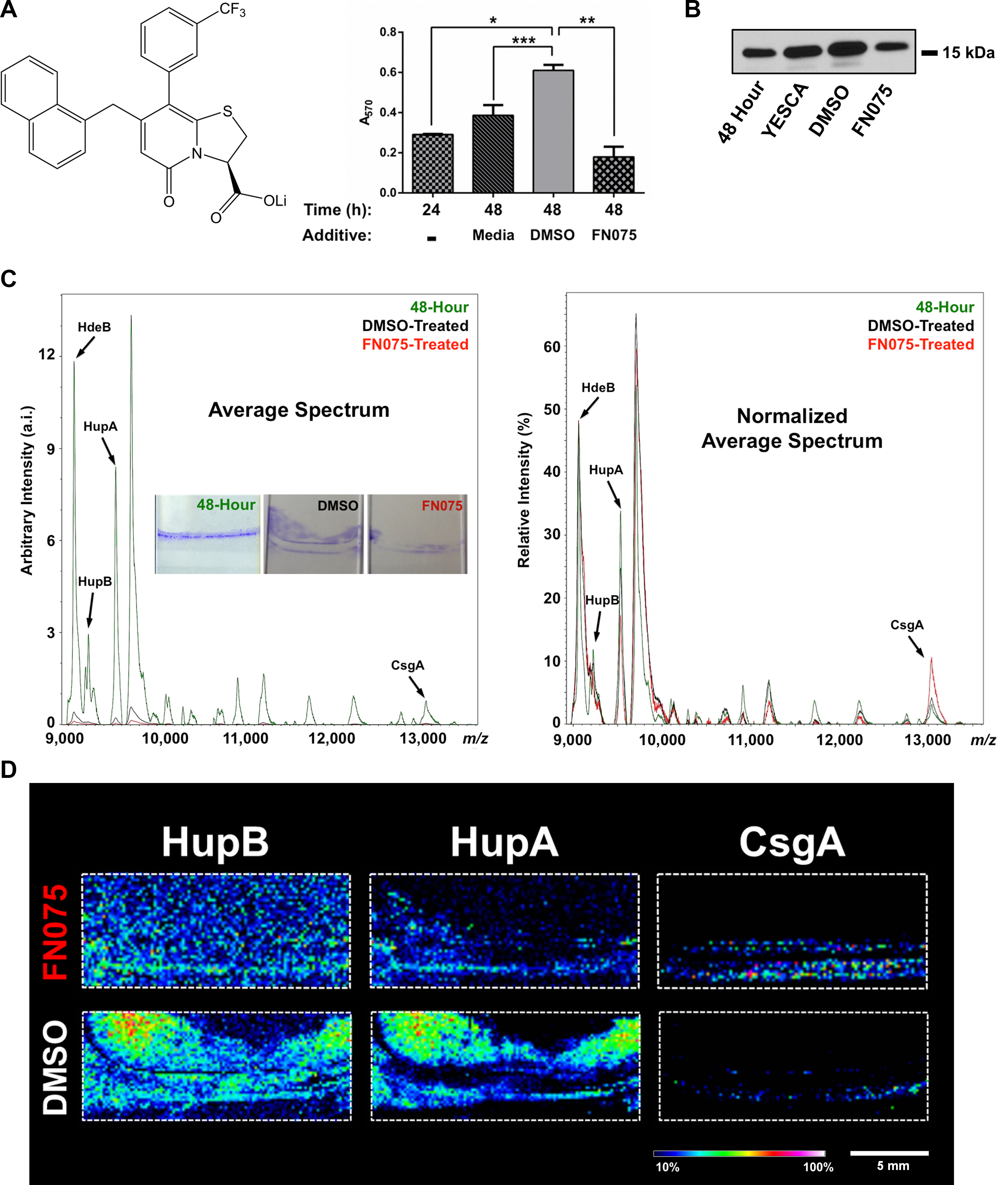

Supplement: S4 Fig — (A) Structure of the compound FN075 and biofilm quantitation for DMSO- and FN075-treated biofilms using crystal violet staining. Data presented as mean with the standard deviation (n = 2 for each condition). Statistical analysis was performed using a two-tailed unpaired Student’s t-test (*p = 0.0037, **p = 0.0089, ***p = 0.0325). (B) Immunoblot for CsgA protein levels between 48-hour cultures, and cultures treated with equivalent volumes of either YESCA media, DMSO (vehicle control), or 125 μm FN075 in DMSO after 24-hours and cultured for another 24-hours. Blot shown is representative of two technical replicates of two biological replicates. (C) Average raw and normalized MALDI IMS spectra from non-treated 48-hour (Green), DMSO-treated (Black), and FN075-treated (Red) biofilm IMS analyses. The average spectra are a result of two biological replicates for each condition after normalization to the total ion current using FlexImaging software. Average spectra were then imported to the mMass Software, background-subtracted and smoothed, and normalized to the most intense ion in the spectra at m/z 7,280. (D) IMS localization and abundance of CsgA within vehicle- or FN075-treated biofilms reveals an increase in distribution throughout the liquid-exposed region of the biofilm. Images shown for each condition are representative of two biological replicates and were processed as described in Fig. 2. Scale bar = 5 mm. (TIF) [file ppat.1004697.s004.tif]

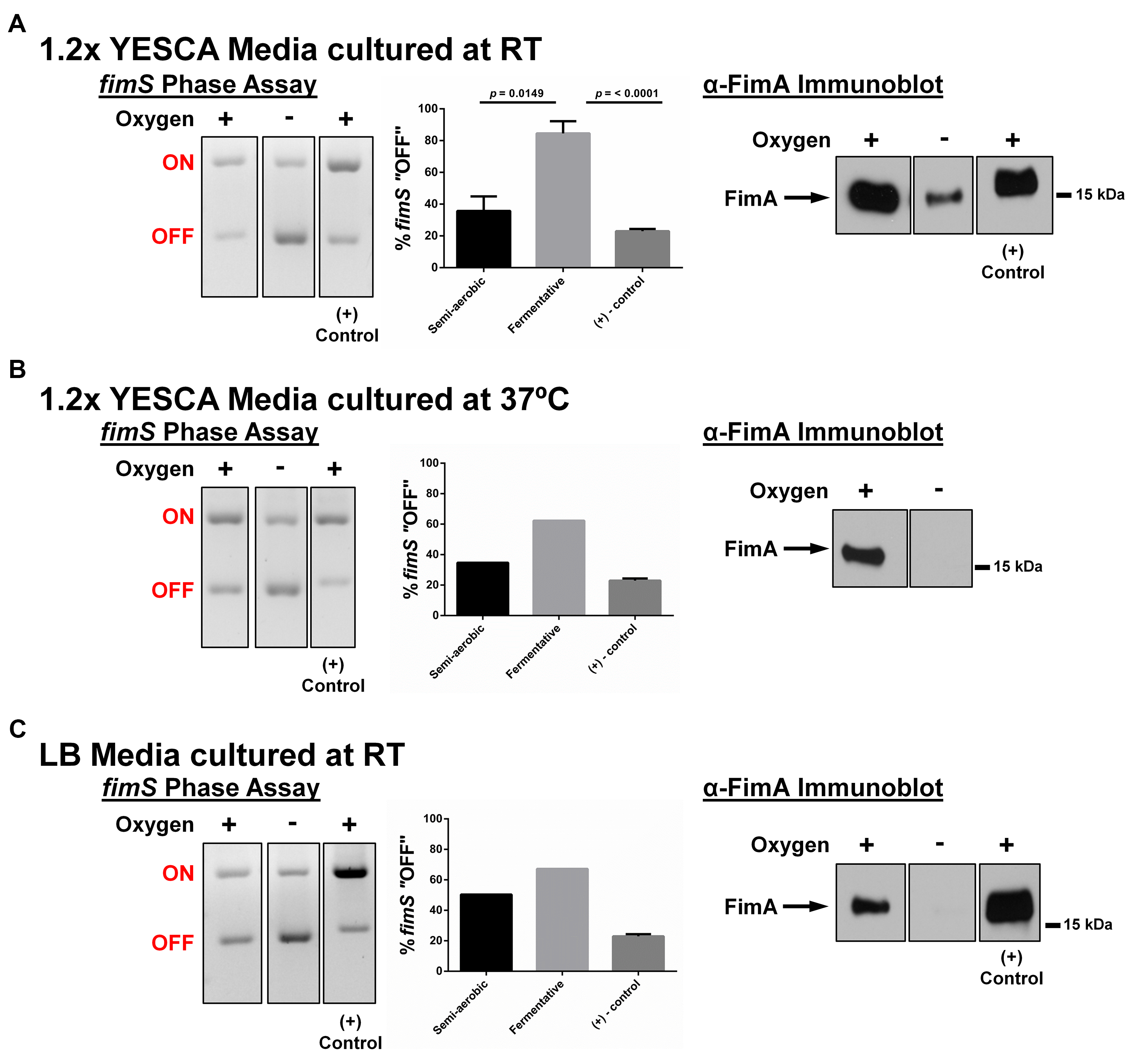

Supplement: S5 Fig — (A) Phase assay and FimA immunoblot analysis of WT UTI89 cultured in 1.2x YESCA media at room temperature under semi-aerobic and fermentative growth conditions. Phase assay quantitation, n = 3. Statistical analysis performed by two-tailed unpaired Student’s t-Test in GraphPad Prism 6, with determined p-values shown. Immunoblot representative of n = 4 analyses. (B) Phase assay and FimA immunoblot analysis of WT UTI89 cultured in 1.2x YESCA media at 37°C under semi-aerobic and fermentative growth conditions. Phase assay quantitation, n = 1. Immunoblot representative of n = 2 analyses. (C) Phase assay and FimA immunoblot analysis of WT UTI89 cultured in LB media (pH 7.4) at room temperature under semi-aerobic and fermentative growth conditions. Phase assay quantitation, n = 1. Immunoblot representative of n = 2 analyses. Statistical analysis of phase quantitation not performed for (B) and (C) due to insufficient number of biological replicates. All data presented as outlined in Fig. 2. All cultures for the analyses in A-C were started from primary overnight cultures grown at 37°C with shaking conditions therefore each population began primarily phase fimOFF. (TIF) [file ppat.1004697.s005.tif]

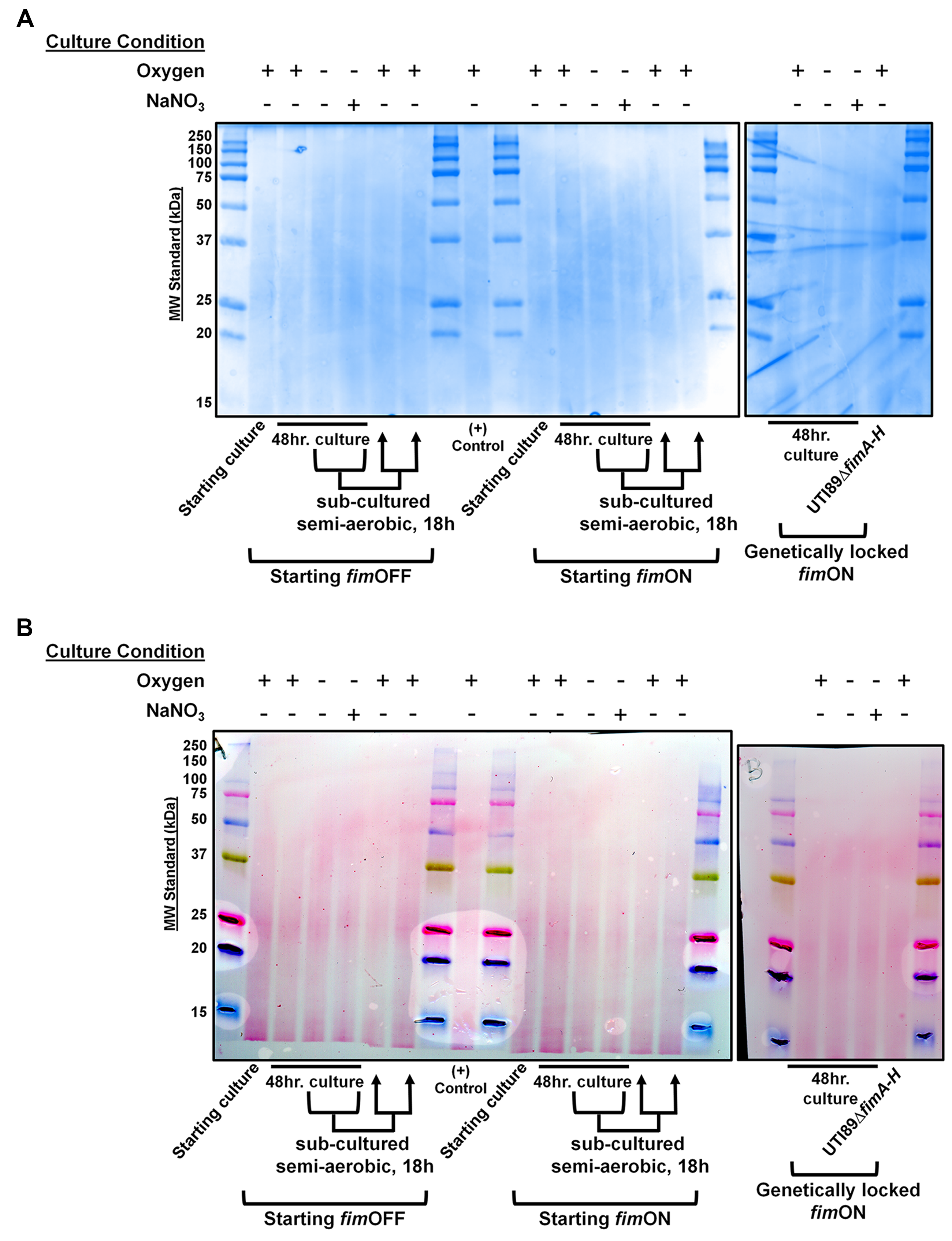

Supplement: S6 Fig — (A) Coomassie stained gels post-transfer for the immunoblots presented in Fig. 5. (B) Ponceau S staining of membranes post-transfer for immunoblots shown in Fig. 5. Together these data show equal loading and protein levels of gels pre-transfer and equal transfer to the membranes used for immunoblotting. (TIF) [file ppat.1004697.s006.tif]

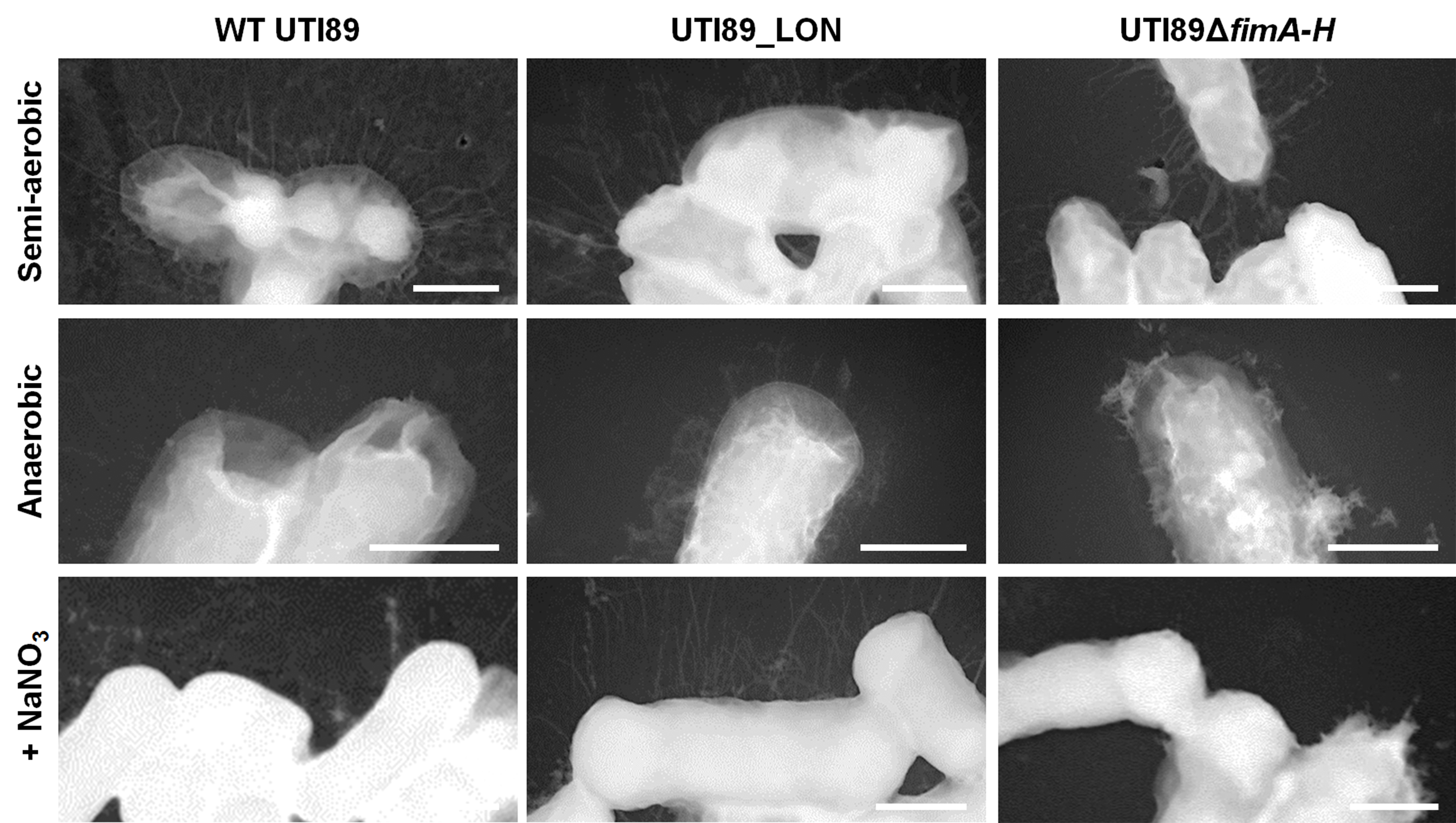

Supplement: S7 Fig — Representative TEM micrographs for each strain under the three tested growth conditions. Cultures were initiated from static overnight stocks and cultured statically for 48 hours in LB media (pH 7.4) at 37°C under the respective growth condition. Scale bar = 500 nm. (TIF) [file ppat.1004697.s007.tif]

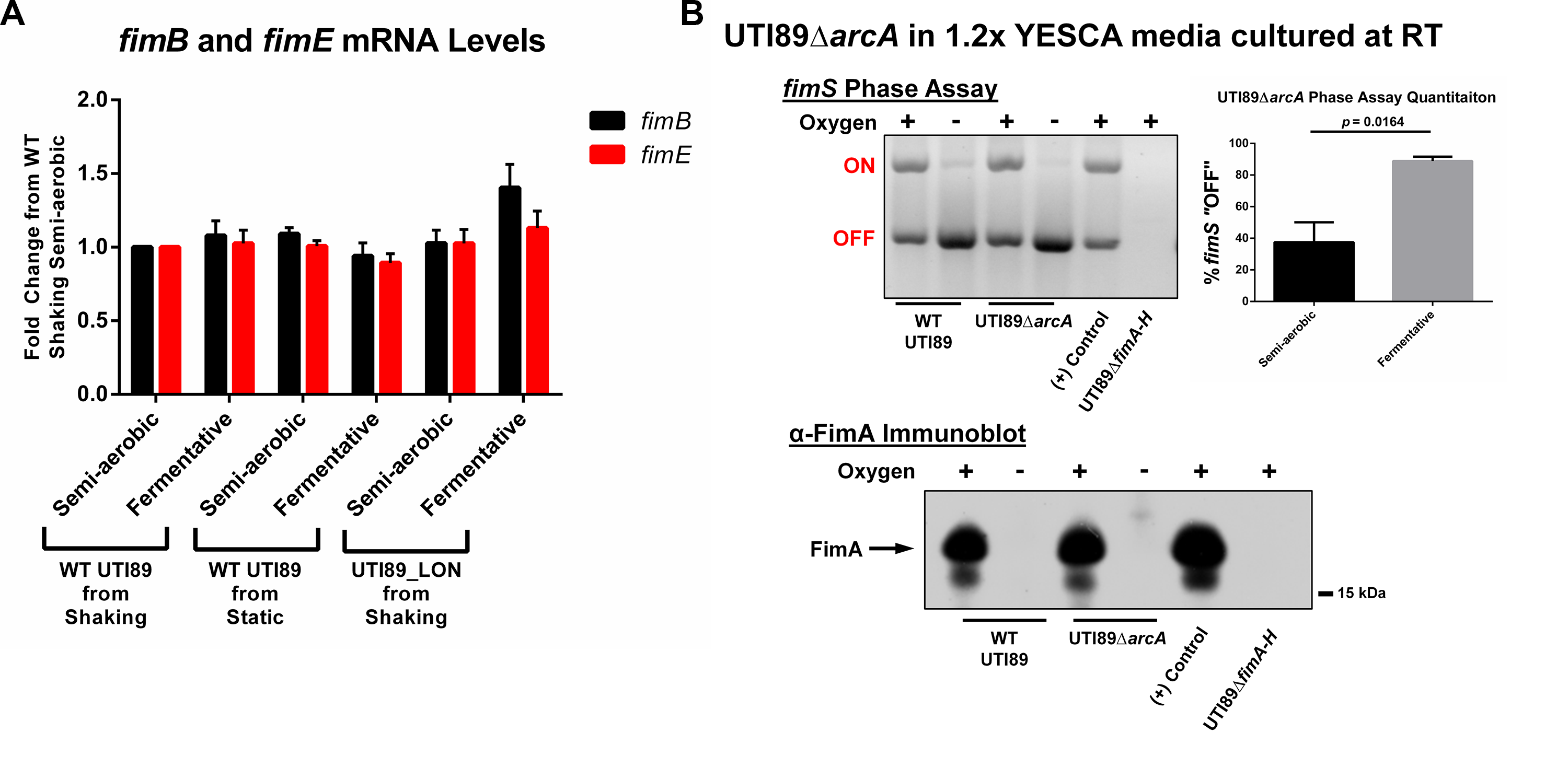

Supplement: S8 Fig — (A) qPCR analysis of fimB and fimE steady-state mRNA transcript levels from semi-aerobic and fermentative cultures started from WT UTI89 populations primarily fimOFF (shaking), WT UTI89 populations primarily fimON (static), or genetically locked fimON (UTI89_LON, started from shaking). RNA was extracted from bacterial pellets obtained from cultures presented in Fig. 5. Data is graphed as the mean ± SEM from two independent biological replicates, with three technical replicates of three different cDNA concentrations used for each biological replicate (9 technical reads per bio replicate). All data are normalized to the internal DNA gyrase (gyrB) values calculated within each sample and presented as the fold-change from WT UTI89 cultures started from primarily fimOFF populations grown under semi-aerobic conditions. Statistical analysis was performed via two-way ANOVA with Bonferroni’s multiple comparisons test. No statistical differences were noted. (B) Phase assay and FimA immunoblot with UTI89ΔarcA indicates this oxygen-dependent regulation of fim expression is not governed by the ArcAB two-component system. Data shown was obtained from cultures starting from populations primarily fimOFF (shaking) and grown in 1.2x YESCA media at room temperature. Phase assay quantitation, n = 3. Statistical analysis performed by two-tailed unpaired Student’s t-Test in GraphPad Prism 6, with determined p-values shown. Immunoblot representative of n = 5 analyses. (TIF) [file ppat.1004697.s008.tif]
