# Supplementary material for: Adhesive Fiber Stratification in Uropathogenic Escherichia coli Biofilms Unveils Oxygen-Mediated Control of Type 1 Pili
Source: PLoS Pathog. 2015 Mar 4;11(3):e1004697. doi: 10.1371/journal.ppat.1004697 (PMC4349694; doi:10.1371/journal.ppat.1004697)
Supplement: S1 Table — Listed are the growth media, temperatures, and oxygen growth conditions under which we examined the expression of type 1 pili from the UPEC strain UTI89. All cultures were grown statically for 48 hours prior to analysis for pili expression. NaNO3, sodium nitrate. (DOCX) [file ppat.1004697.s010.docx]

**Table S1. Growth media and conditions used for analysis of type 1 pili expression in UPEC.**

| Media | Temperature | Oxygen Condition |
| --- | --- | --- |
| 1.2x Yeast-Extract Casamino Acids (YESCA) | Room Temperature | Semi-aerobic |
|  | Room Temperature | Fermentative |
|  | Room Temperature | Anaerobic + 40mM NaNO_3_ |
|  | 37°C | Semi-aerobic |
|  | 37°C | Fermentative |
|  | 37°C | Anaerobic + 40mM NaNO_3_ |
| Luria-Bertani (LB) – pH 7.4 | Room Temperature | Semi-aerobic |
|  | Room Temperature | Fermentative |
|  | Room Temperature | Anaerobic + 40mM NaNO_3_ |
|  | 37°C | Semi-aerobic |
|  | 37°C | Fermentative |
|  | 37°C | Anaerobic + 40mM NaNO_3_ |
