# Supplementary material for: Adhesive Fiber Stratification in Uropathogenic Escherichia coli Biofilms Unveils Oxygen-Mediated Control of Type 1 Pili
Source: PLoS Pathog. 2015 Mar 4;11(3):e1004697. doi: 10.1371/journal.ppat.1004697 (PMC4349694; doi:10.1371/journal.ppat.1004697)
Supplement: S1 Methods — (DOCX) [file ppat.1004697.s012.docx]

**Supplemental Materials and Methods:**

**Matrix Application for Bacterial Biofilm MALDI-TOF IMS Analysis**

A matrix of recrystallized 15 mg/mL 2,5-Dihydroxybenzoic acid (DHB) (Fisher Scientific) and 5 mg/mL a-Cyano-4-hydroxycinnamic acid (CHCA) (Sigma Alderich Chemical Company) was prepared in 90% HPLC-grade acetonitrile (Fisher Scientific) with 0.2% trifluoroacetic acid (TFA) (Sigma-Alderich). Matrix was applied to dry films using a TM-Sprayer (HTX Imaging). Matrix was applied with 8 passes of the TM-Sprayer in an offset/overlay pattern with 2 mm spacing at 1200 mm/min, using a solvent of 90% acetonitrile (ACN) flowed at 0.2 mL/min at a nozzle temperature of 100°C, utilizing nitrogen as an inert nebulizing gas. Spray passes were offset and rotated to ensure homogenous sample coverage. Samples were rehydrated in an oven at 85°C to facilitate analyte crystallization with matrix. Samples were warmed for 2 minutes in the oven, and then sealed in a rehydration chamber with 1 mL of 10% acetic acid (AcOH) for 2.5 minutes.

**MALDI-TOF IMS Parameters**

Samples were analyzed using a Bruker Autoflex Speed mass spectrometer (Bruker Daltonics) equipped with a Nd:YAG (355 nm) Smartbeam laser operated in linear positive-ion mode. Images were obtained at 150 micron (µm) lateral resolution. Laser repetition rate was 1 KHz. Two hundred laser shots were collected at each pixel in 50 shot increments in a random walk pattern. Ion extraction voltage was 19.17 kV and ion acceleration voltage was 17.90 kV. Ions were collected with a delayed extraction time of 350 ns and a lens voltage of 6.30 kV. Ions under *m/z* 2000 were suppressed. A mass range of *m/z* 2,000- 25,000 was collected. The detector gain was 3,400 volts.

**Protein Fractionation and Identification for UPEC Biofilms**

UPEC biofilms were lysed under one of the following conditions; 88% formic acid (FA), 35% AcOH, 50% ACN / 0.2% TFA, or 0.5 µg/mL lysozyme in TE Buffer (pH 8.0). Lysates obtained under the same lysis condition were pooled together and sonicated for 15 minutes in a room-temperature water bath, and then centrifuged at 14,000 x g and 4°C for 15 minutes. The supernatant was then transferred to a new 1.5mL microfuge tube, and dried under vacuum centrifugation using a SpeedVac Vacuum Concentrator (Thermo Scientific).

Extracts were fractionated using reversed-phase (RP) high performance liquid chromatography (HPLC). RP-HPLC fractionation was performed using a Waters 2690 (Alliance) HPLC (Milford, MA) with a 2478 Dual Wavelength Absorbance Detector (Waters Corporation), and a Gilson FC 203B 96-well plate fraction collector. UPEC lysates were fractionated on either a Vydac 208TP series 208TP5315, 150 x 3.2mm 5µ C8 column fitted with a Vydac 208TP C8 7.1x4.6mm guard column (Grace Vydac), or an Aeris Widepore 150 x 2.1 mm 3.6µ C18 column fitted with a SecurityGuard ULTRA UHPLC Widepore C18 guard cartridge (Phenomenex. Solvent A was 100% H_2_O with 0.1% TFA, and Solvent B was 100% ACN with 0.1% TFA. Fractionation was performed at a flow-rate of 0.5mL/min, with fractions collected in 1-minute intervals into a 96-well plate, and elution off of the column was monitored at 214 and 280nm. The column was equilibrated with 5% Solvent B, and 200µL of the lysate was loaded onto the column and washed with 5% Solvent B for 10 minutes. The elution was performed with a gradient of 5-25% Solvent B from 10-15 minutes, 25-60% Solvent B from 15-65 minutes, and then 60-95% Solvent B from 65-75 minutes. The column was then washed with 95% Solvent B, and re-equilibrated back to 5% Solvent B. After fractionation, 96-well plates were dried using the Speedvac vacuum concentrator, and dried samples were stored at 4ºC until MALDI-TOF MS analysis.

Fractions were reconstituted in a minimal volume of 60% ACN / 0.2% TFA, and a MALDI spectrum was acquired from each fraction. Fractions containing *m/z* species of interest were selected for in-solution tryptic digestion for protein identification. Fractions were extracted from the 96-well plate and dried. Samples were resuspended in 100 mM Tris-HCl (pH 7.8) / 8 M urea, and disulfide bonds were then reduced with 10 mM dithiothreitol (DTT) (Sigma-Aldrich, St. Louis, MO) for 1 hour at 37°C. After reduction, samples were alkylated with 55 mM iodoacetamide (IA) for 30 minutes at room temperature in the dark. The concentration of urea was the reduced to 2mM with the addition of 100 mM Tris-HCl (pH 7.8), and 500 ng of trypsin (Promega or Sigma-Aldrich) was added to each sample and incubated overnight at 37°C. After overnight digestion, the reaction was stopped with the addition of 1 µL of 100% AcOH. Digested fractions were submitted to the Vanderbilt University Mass Spectrometry Research Center Proteomics Core for LC-MS/MS identification.

Resulting peptides were analyzed by a 70-minute data dependent LC-MS/MS analysis.  Briefly, peptides were auto-sampled onto a 200 mm by 0.1 mm (Jupiter 3 micron, 300A), self-packed analytical column coupled directly to an LTQ (ThermoFisher) using a nanoelectrospray source and resolved using an aqueous to organic gradient.  A series of a full scan mass spectrum followed by 5 data-dependent tandem mass spectra (MS/MS) was collected throughout the run and dynamic exclusion was enabled to minimize acquisition of redundant spectra.  MS/MS spectra were searched via SEQUEST against the Escherichia coli (strain UTI89 / UPEC) (taxon identifier 364106) database that also contained reversed version for each of the entries. ([http://www.ncbi.nlm.nih.gov/pubmed/7741214](http://www.ncbi.nlm.nih.gov/pubmed/7741214)).  Identifications were filtered and collated at the protein level using Scaffold (Proteome Software).
